# Fluorescence turn on amine detection in a cationic covalent organic framework

**DOI:** 10.1038/s41467-022-31393-2

**Published:** 2022-07-07

**Authors:** Gobinda Das, Bikash Garai, Thirumurugan Prakasam, Farah Benyettou, Sabu Varghese, Sudhir Kumar Sharma, Felipe Gándara, Renu Pasricha, Maria Baias, Ramesh Jagannathan, Na’il Saleh, Mourad Elhabiri, Mark A. Olson, Ali Trabolsi

**Affiliations:** 1 Chemistry Program, New York University Abu Dhabi (NYUAD), Saadiyat Island, Abu Dhabi, UAE; 2 NYUAD Water Research Center, New York University Abu Dhabi (NYUAD), Saadiyat Island, Abu Dhabi, UAE; 3 CTP, New York University Abu Dhabi (NYUAD), Saadiyat Island, Abu Dhabi, UAE; 4 Engineering Division, New York University Abu Dhabi (NYUAD), Saadiyat Island, Abu Dhabi, UAE; 5 Materials Science Institute of Madrid – CSIC, Sor Juana Inés de la Cruz 3, 28049 Madrid, Spain; 6 Chemistry Department, College of Science, United Arab Emirates University, P.O. Box 15551, Al Ain, UAE; 7 National Water and Energy Center, United Arab Emirates University, P.O. Box 15551, Al Ain, UAE; 8 Université de Strasbourg, Université de Haute-Alsace, CNRS, LIMA, UMR 7042, Equipe Chimie Bioorganique et Médicinale, ECPM, 25 Rue Becquerel, 67000 Strasbourg, France; 9 Department of Physical and Environmental Sciences, Texas A&M University Corpus Christi, 6300 Ocean Dr., Corpus Christi, TX 78412 USA

**Keywords:** Polymers, Organic molecules in materials science, Synthesis and processing

## Abstract

Ionic covalent organic frameworks (iCOFs) are new examples of porous materials and have shown great potential for various applications. When functionalized with suitable emission sites, guest uptake via the ionic moieties of iCOFs can cause a significant change in luminescence, making them excellent candidates for chemosensors. In here, we present a luminescence sensor in the form of an ionic covalent organic framework (TGH^+^•PD) composed of guanidinium and phenanthroline moieties for the detection of ammonia and primary aliphatic amines. TGH^+^•PD exhibits strong emission enhancement in the presence of selective primary amines due to the suppression of intramolecular charge transfer (ICT) with an ultra-low detection limit of 1.2 × 10^‒7^ M for ammonia. The presence of ionic moieties makes TGH^+^•PD highly dispersible in water, while deprotonation of the guanidinium moiety by amines restricts its ICT process and signals their presence by enhanced fluorescence emission. The presence of ordered pore walls introduces size selectivity among analyte molecules, and the iCOF has been successfully used to monitor meat products that release biogenic amine vapors upon decomposition due to improper storage.

## Abstract (web summary)

Ionic covalent organic frameworks (iCOFs) are new examples of porous materials and show great potential for various applications. Here, the authors demonstrate functionalization of an iCOFs with suitable emission sites and application as chemosensor for amine detection with high sensitivity which can be used to monitor meat spoilage.

## Introduction

Selective detection of hazardous chemicals is a critical factor in limiting their potential release into the environment and preventing consequential damages^1,2^. Therefore, the development of new materials that can detect and capture hazardous chemicals is an important area of scientific research^3–5^. In particular, fluorescence-based sensing materials have attracted considerable attention and offer significant advantages over non-emissive sensors^1,6,7^, such as fast response time^8^, high sensitivity^9,10^, and naked-eye detection^11^. In the development of such systems, improving the sensor–analyte interaction is a major challenge to achieve the desired efficiency. However, such a challenge could be potentially addressed by using porous materials^12–18^ whereby the ordered pores allow diffusion of analytes to improve their interaction with the reactive components through large areas of the pore surfaces.

Ionic covalent organic frameworks (iCOFs)^19–24^ are a class of extended crystalline organic solid architectures with ionic moieties as structural units. The remarkable chemical stability and tunable surface properties of iCOFs make them a suitable choice for various applications^25–28^. While there are numerous reports of neutral frameworks^29–36^, COFs with charged backbones and structural units are rarely reported^19,23,25,26,28,37^. The introduction of ionic motifs in COFs has increased their application potential for micropollutant removal^19,38,39^, catalysis^40,41^, and molecular separation^39^ through their enhanced interaction with pore surfaces. Here, the ionic motifs create ionic channels within the network that enabled enhanced molecular recognition with incoming guest molecules through electrostatic host-guest interactions^19,38^. In this way, the application of fluorescent iCOFs as efficient molecular sensors^26,42^ is quite promising; however, the use of such structures for molecular recognition signaled by luminescence remains relatively unexplored^26^. Among the reported ionic building blocks^43–45^ for the construction of iCOFs, guanidinium^25,46^ is a representative entity that belongs to the Lewis-acid class of organic compounds. On account of its electron deficiency, guanidinium introduces π-electron deficiency into the framework, increasing the interaction of the material with electron-rich species^26^. To take advantage of this interesting phenomenon, we hypothesized that the incorporation of this functional group into a covalent organic framework would create multiple acidic sites throughout the network, leading to the ability to detect alkaline amines through acid–base interactions.

The detection of volatile organic amines is an important but challenging task, and there is a significant need for the development of a suitable chemical sensor that can detect the presence of amines of interest at trace levels^6,47^. Organic amines are released into the environment through their use in the dye^48^, fertilizer^49,50^, and pharmaceutical industries^51,52^. To prevent the negative environmental impact of amine-based wastes, trace levels of these amines must be efficiently detected. Another valuable application of amine detection is the monitoring of amine content in fresh foods to track food decomposition^52,53^. For example, amine-based molecules such as cadaverine (i.e. 1,5-diaminopentane) or putrescine (i.e. 1,4-diaminobutane) are formed during the decomposition of meat, and therefore the detection of such amines in stored meat products is essential for determining their freshness^49,54^. For such applications, naked-eye detection and portability of the sensing system are major advantages; therefore, fluorescence-based sensors are the most suitable^6,47,55,56^. To date, several luminescent COF-based chemosensors have been developed for the detection of 2,4,6-trinitrophenol^18,57^, other polynitroaromatics^58^, biomolecules^42,59–62^, and heavy metal ions^63,64^. In many of these cases, the emission property is quenched by interaction with the analyte. These materials are referred to as quenchable or “turn off” emission sensors. Such quenching often interferes with other components of the system and makes it difficult to measure emission at high analyte concentrations and to indicate the presence of the analyte of interest. Switchable “Turn on” emission sensors, on the other hand, exhibit enhancement of luminescence properties in the presence of the analyte and are therefore much more suitable for the desired application as a chemosensor. In one report^12^, the applicability of a COF material as an ammonia chemosensor was demonstrated, although its emission turn-off mechanism limited its scope, as mentioned earlier. However, COF-based fluorescent “turn on” sensors for amine detection have not yet been reported.

**Fig. 1:**
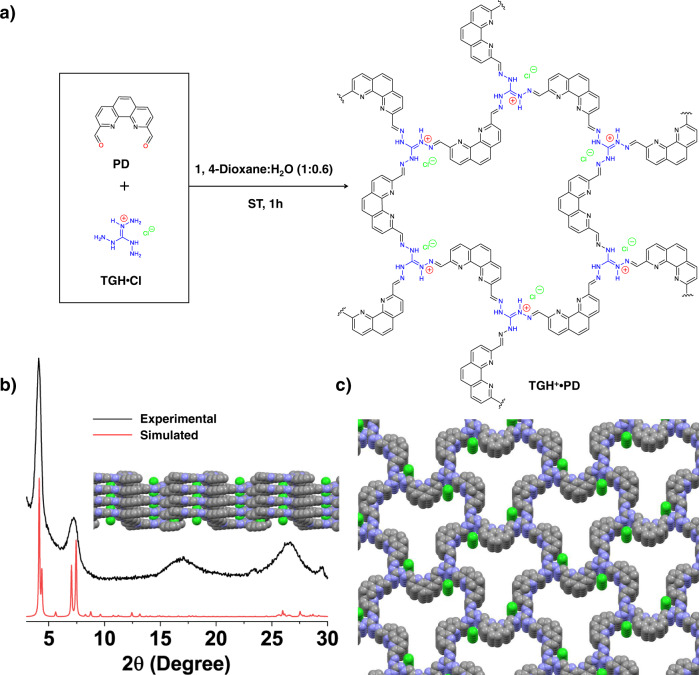
Synthetic strategy for the preparation of iCOF materials. **a** Synthesis and chemical structure of TGH^+^•PD obtained under solvothermal (ST) conditions at 120 °C from a 1:0.6 (v*:*v) 1,4-dioxane:H_2_O mixed solvent. **b** Structural characterization of TGH^+^•PD showing comparison between the experimental powder X-ray diffraction (PXRD) pattern of TGH^+^•PD and the simulated PXRD pattern of the AA stacking model (shown inset). **c** Top view of a space-filling representation containing four stacked layers of TGH^+^•PD.

Here, we report the synthesis of a iCOF-based chemosensor (hereafter referred to as TGH^+^•PD, Fig. 1a) obtained from the condensation of triamino-guanidinium hydrochloride salt (TGH•Cl) and phenanthroline-2,9-dicarbaldehyde (PD). While the presence of the cationic core made TGH^+^•PD highly dispersible in water, the existence of intramolecular charge transfer (ICT) from the phenanthroline (e^−^ donor) to the guanidinium unit (e^−^ acceptor) resulted in an orange emission. This ICT process itself is sensitive to binding events with appropriate analytes that disrupt the electrostatic interaction between the donor–acceptor pair. The net result is a detectable change in the emission of the material. The obtained cationic iCOF can serve as a luminescent and colorimetric probe for the detection of strongly basic organic amines.

## Results

### Synthetic protocol for TGH^+^•PD

The ionic COF (TGH^+^•PD) was prepared by co-condensation of TGH•Cl (42.3 mg, 0.30 mmol) and PD (106.2 mg, 0.45 mmol) in 2 mL of a 1,4-dioxane: H_2_O (1:0.6, v:v) mixed solvent (Fig. 1a). The synthesis was carried out under solvothermal conditions in a 25 mL high-pressure flask equipped with a vacuum valve. Heating the reaction mixture at 120 °C for one hour resulted in the precipitation of an orange solid, which was purified by washing with 1,4-dioxane and ethanol and then dried at 110 °C for 12 h. The resulting product was completely insoluble in water and common organic solvents such as ethanol, dichloromethane, and 1,4-dioxane. Therefore, all structural characterizations of the product were carried out in the solid state.

### Characterization of TGH^+^•PD

**Fig. 2:**
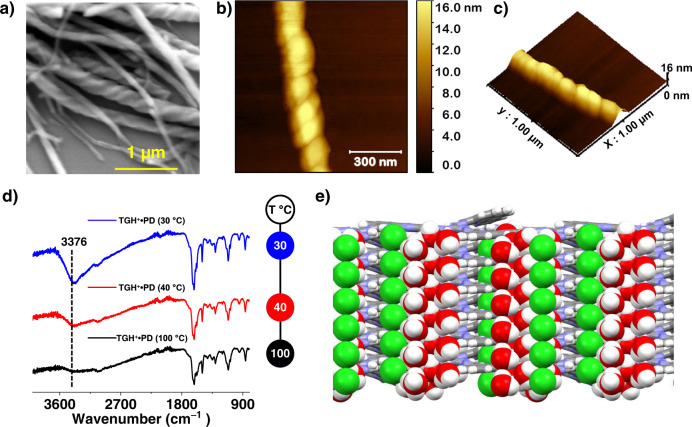
Microscopic characterization of TGH^+^•PD. **a** SEM, **b** AFM, and **c** 3D AFM images of the TGH^+^•PD-based helical fibers. **d** Superimposed variable-temperature FTIR spectra of TGH^+^•PD showing the disappearance of water molecules upon heating. **e** Perspective view of the modeled COF layers along the screw axes illustrating the interactions between counterions, water molecules, and the COF layers.

FTIR spectroscopic analysis (Fig. 2d and Supplementary Fig. 1) of the product revealed the absence of characteristic peaks corresponding to the starting materials (the primary amine moiety of TGH^+^ at ~3200 cm^−1^ and the aldehyde group of PD at 1693 cm^−1^), indicating that the starting materials were consumed during the reaction. However, because of the reversible nature of the COF formation reaction, 100% conversion of the precursors into crystalline COF is not possible. At the end of the reaction, the reaction mixture contains some oligomers and some unreacted precursors in addition to the precipitated COF materials. During the purification step, we removed those soluble species by washing with different solvent combinations and therefore, as expected, the yield (57%) is less than 100%. A broad peak was observed at 3376 cm^−1^ which was attributed to hydrogen-bonded water molecules trapped in the framework. This band disappeared completely when the compound was heated above 100 °C and reappeared upon cooling to room temperature in ambient air (Supplementary Fig. 2). The role that these hydrogen-bonded water molecules played in affecting the morphological features of the framework will be discussed in a later section. The FTIR spectrum also displayed a stretching band signal at 1619 cm^−1^ that was attributed to the newly formed imine bond between the amine and the aldehyde.

The formation of the imine bond was further confirmed by solid-state ^13^C cross-polarization magic-angle spinning (CP/MAS) NMR spectra (Supplementary Fig. 3), in which a characteristic peak for the imine C is observed at ∼147 ppm. Another distinct peak at 152 ppm of the NMR spectrum corresponded to the C atom of the guanidinium unit.

The formation of the network structure and its high thermal stability were clearly observed in TGA. From the TGA diagram (Supplementary Fig. 4), the initial weight loss of 10%, which occurs between 50 and 120 °C is due to the removal of weakly adsorbed water. The subsequent weight loss of 20% observed up to 200 °C corresponds to the gradual release of hydrogen-bonded water molecules adsorbed in the pores. TGA studies showed the release of ~10 water molecules per unit cell of the framework structure. The relatively higher thermal stability of the material compared to the starting materials confirmed the formation of the structural network, the porosity of which was analyzed by gas adsorption. N_2_ gas adsorption isotherm at 77 K (Supplementary Fig. 5) showed a limited uptake capacity for N_2_ gas molecules. The positively charged pores created by the small guanidinium cores most likely restricted the adsorption of nitrogen molecules, which have a larger kinetic diameter, and resulted in a relatively low Brunauer–Emmett–Teller surface area of 16 m^2^ g^−1^. The observed low porosity is mainly due to the short length of the linkers, in addition to the presence of chloride ions as counter anions and water molecules within the pore channels. This limitation of low porosity for guanidinium-based COFs is well established^28,43,46,65^.

### Structural analysis of TGH^+^•PD

The two most intense diffraction peaks are observed at 2*θ* = 4.1°, and at 2*θ* = 7.2° (Fig. 1b), with a broader peak centered at a higher 2*θ* value of 26.9°. Using this powder X-ray diffraction (PXRD) pattern, and considering the geometry of the organic linkers (C_2_ + C_3_ combination), a crystal model based on the formation of honeycomb layers (**hcb**) was first constructed. Starting from the model with maximum symmetry embedding of stacked **hcb** layers in the hexagonal *P*−6*m*2 space group, new crystal models were built by reducing the symmetry to space groups compatible with the experimentally observed chirality of the sample (see below), and the position of the diffraction lines. The crystal models were then geometrically optimized by energy minimization procedures with the use of a density functional-based tight-binding method, as implemented in the DFTB+ module of *Materials Studio Software*. A crystal structure was thus simulated in the monoclinic *P*2_1_ space group, with lattice parameters of *a* = 41.44 Å, *b* = 25.11 Å, *c* = 3.49 Å, *β* = 103.31°, where the layers are parallel to the *ab* plane (Fig. 1c). Chloride ions were then introduced into the pores to balance the positive charge of the layers. According to this crystal model, the experimentally observed diffraction line at 2*θ* = 4.1° corresponds to the (110) and (200) planes, while the peak at 7.2° is composed of the (020) and (310) planes.

The observed diffraction peaks in the experimental PXRD pattern are rather broad, indicative of limited crystallinity. This is comparable to what is commonly observed for 2D COF samples, where the interlayer interactions are not covalent. Considering the limited information in the X-ray diffraction pattern, the structural analysis process is completed through computer modelization, the proposed crystal model is the one with the best agreement between simulated and experimental pattern. Moreover, the limited crystalline peaks in our iCOFs is due to strong charge repulsion between adjacent cationic sites. Peak broadening in the wide-angle region can also be attributed to the limited size of the crystalline domains. In Fig. 1b, we added the calculated pattern for a perfect crystal model, to clearly show the main diffraction peak positions and their relative intensities. However, if a small (25 nm) crystallite size is introduced in the pattern calculation, the diffraction peaks are significantly broadened, and in good agreement with the experimental pattern, as shown in the (Supplementary Fig. 6). On the other hand, the broad area between 2*θ* = 15°−20° observed in the experimental pattern is due to the experimental acquisition conditions. In particular, due to the limited sample amount and its weak scattering power, a broad feature appears arising from scattering by the sample holder. To further confirm this point, we collected an additional PXRD pattern covering the entire illuminated area of the holder by mixing the COF with alumina powder. In the resulting powder pattern, the COF peaks are visible along with the alumina narrow diffraction signals, but now the broad area in the 15–20 region is no longer present (Supplementary Fig. 7). All the additional spectroscopic evidences strongly suggest the formation of an extended structure, and the observed diffraction peaks clearly indicated the presence of long-range order, and were in good agreement with the proposed crystal models. While the exact interlayer stacking sequence (if any) cannot be unambiguously determined with X-ray diffraction data, for the sake of comparison, the simulated patterns for additional crystal models were made based on different stacking modes of equivalent *hcb* layers (Supplementary Fig. 8), and showed the differences in peak position and relative intensity. To gain more structural information we successfully exfoliated TGH^+^•PD COF from its aqueous suspension, using high-energy probe sonication for one hour. The exfoliated material was comprised of nanosheets that were a few atomic layers thick, as confirmed by scanning electron microscopy (SEM), high-resolution transmission electron microscopy (HRTEM), and atomic force microscopy (AFM) analysis (Supplementary Fig. 9). At high magnification, HRTEM showed that the COF consisted of stacked layers of 2D sheets (Supplementary Fig. 9b–d), which indicated a highly ordered alignment and corresponded to the (001) plane (Supplementary Fig. 9c, d). The *d*-spacing obtained from HRTEM (0.36 nm) matched well with the X-ray diffraction (XRD) profile, whereas Supplementary Fig. 9d revealed the porous texture of TGH^+^•PD. The crystallinity of the iCONs was also confirmed by its selected area electron diffraction (SAED, Supplementary Fig. 9e) pattern, which exhibited distinct electron diffraction spots, which corresponded to the (001) and (440) planes of TGH^+^•PD COF with *d*-spacing of ~3.3 Å, and ~2.4 Å, respectively matched closely to the simulated PXRD pattern. The stacked sheet-like morphology was further confirmed by AFM analysis (Supplementary Fig. 9f), whereby the sheet thickness was found to be ~3–4 nm. Based on the AFM data, we determined that TGH^+^•PD COF comprised of about ten stacked layers. The morphological features of the exfoliated material are consistent with other 2D-covalent organic nanosheets obtained using guanidium linkers^25,28^.

### Morphological analysis and their formation mechanism

Helical fibrillar morphological features were observed on SEM images of TGH^+^•PD (Fig. 2a and Supplementary Fig. 10). Detailed analyses of different areas of the sample showed that both P and M helices were present in the resulting material. AFM in tapping mode measured an average width of 120 nm for the helices with a step length of 110 nm (Fig. 2b, c). The origin of helicity can be attributed to the water molecules causing hydrogen bonding between the layers. The water molecules present in the framework formed a helical chain and acted as a template for further nucleation. As a result, the phenanthroline cores align through hydrogen bonds, leading to the observed helical morphology. The presence of water molecules was confirmed by FTIR spectroscopic analysis (Fig. 2d and Supplementary Fig. 1) and TGA (Supplementary Fig. 4), as mentioned previously. Moreover, the role of water molecules in the formation of the helical morphology was verified by SEM after the water molecules were removed from the system following heating (Supplementary Fig. 11). First, the uniform helical morphology of the original samples exhibited damage after heat treatment. The helical morphology of the material was then restored when exposed to water (Supplementary Fig. 11c), confirming the role of water in this process. Second, when the synthesis was carried out under identical conditions but using anhydrous 1,4-dioxane as solvent, the resulting material (TGH^+^•PD-anh) consisted of hollow tubes with no evidence of helical morphology (Supplementary Fig. 12). These observations indicate the importance of water molecules in the formation of helical fibers. Further insight into the formation of the twisted fibers was gained through a simulated annealing process that described the possible location and interactions of water molecules within the structure. The results of the simulation study showed that the water molecules form a hydration sphere around the chloride ions, which are mainly located near the guanidinium units through ionic interaction. On account of the stacking of the phenanthroline moieties, this hydrogen-bonded network propagated along the crystallographic *c* axis (Fig. 2e). This arrangement is similar to a related guanidinium-based molecular compound that also crystallizes and forms helical fibers^66^. It can be concluded that the hydrogen bonds between the water molecules and the chloride counterions of adjacent layers cause a twisting action during fiber growth along the layer stacking direction.

We have performed variable temperature PXRD at 25 and 100 °C to monitor any effect of the water molecules on the material’s crystallinity. However, we did not observe any change in the peak position in the low-angle region. Interestingly, in the wide-angle region, we found a noticeable shift from 2*θ* = 26.9° to 25.5° of the PXRD peak assigned to the (001) plane (Supplementary Fig. 13). Since the first peak remains unaffected by the water elimination, the network structure of TGH^+^•PD is observed to stay unchanged by heating. A complete color change from orange to dark red indicated the complete loss of hydrogen-bonded water molecules from the pores, which was confirmed by FTIR (Supplementary Fig. 2). The COF material synthesized from anhydrous 1,4-dioxane (TGH^+^•PD-anh) exhibited a similar PXRD profile (Supplementary Fig. 14) and porosity (Supplementary Fig. 15) as TGH^+^•PD, synthesized from an ethanol and water (2:1, v/v) mixed solvent. However, the absence of the water template prevented the formation of a helical morphology as seen by SEM. Instead, a hollow-tubular morphology was observed. Moreover, analysis of N_2_ adsorption revealed a limited BET surface area (12 m^2^/g), like that of TGH^+^•PD.

Circular dichroism (CD) was also used to further quantify the optical properties of TGH^+^•PD generated by helicity (Supplementary Fig. 16). The COF suspension in 1:1, v:v 1,4-dioxane:H_2_O mixed solvent, did not produce any CD activity under a variety of conditions tested. This was caused by the presence of a racemic mixture containing both P and M helical fibers in the suspensions, neutralizing the contribution of each optical rotation. However, when the same suspension was drop-casted on a quartz plate to form a film (20 mm diameter × 1 mm thick), TGH^+^•PD showed CD signals with positive and negative Cotton effects with a dominant peak centered at 372 nm (Supplementary Fig. 16a), which is comparable to their UV−V is absorption maximum centered at 381 nm (Supplementary Fig. 16b). Additionally, the recorded CD spectra of different batches of the same sample showed the random appearance of positive CD signals and negative CD signals (Supplementary Fig. 17). This observation clearly indicates that chirality is randomly distributed in the films and there is no excess of one chiral handedness over the other.

### Photophysical property and Fluorescent amine sensing

**Fig. 3:**
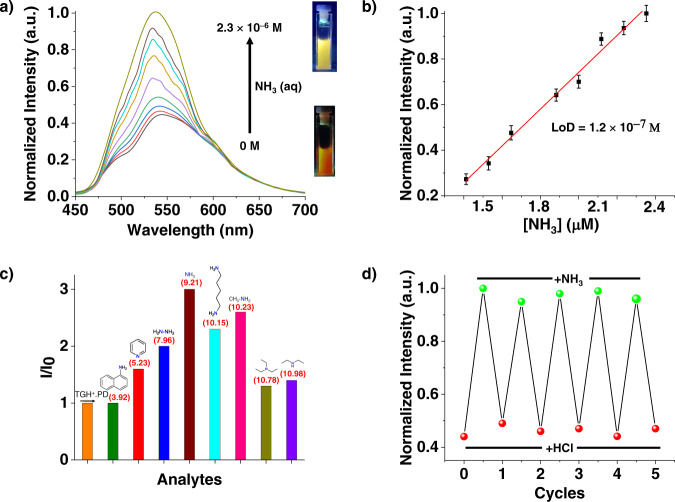
Amine responsive behavior of fluorescent TGH^+^•PD COF. **a** Change in the emission of an aqueous dispersion of TGH^+^•PD (*λ*_ex_ = 365 nm) with increasing amounts of NH_3_ (aq), inset: photographs of an aqueous dispersion of TGH^+^•PD under UV lamp irradiation showing the changes in fluorescence after the addition of NH_3_ (aq). **b** Linear fit to the plot of emission against the concentration of NH_3_ (0–2.3 × 10^−6^ M) added to an aqueous dispersion of TGH^+^•PD to calculate the limit of detection (LoD) for NH_3_, the error bars represent the s.d. of triplicate measurements. **c** Bar diagram representing the relative fluorescence intensity of TGH^+^•PD in the presence of various amines, where I_o_ is the intensity of initial TGH^+^•PD, and I is the final intensity in response to the analyte (the numbers in parentheses indicate the corresponding p*K*a value). **d** Reversible change in fluorescence intensity of TGH^+^•PD upon an alternate addition of NH_3_ and HCl in water (*λ*_ex_ = 365 nm).

The ionic nature of TGH^+^•PD makes it readily dispersible in water and so we have studied its photophysical properties in an aqueous suspension. The emission spectrum of an aqueous solution of TGH^+^•PD consists of an orange emission band centered at 553 nm (*λ*_ex_ = 365 nm, Fig. 3a), which was attributed to the ICT from phenanthroline to the guanidium moiety. ICT fluorescent probes involve an overall charge distribution throughout the network and show a significant change in fluorescence upon binding with analytes^67^.

Inspired by the electron-deficient and Lewis acidic nature of TGH^+^•PD, we investigated its potential as a chemosensor for the detection of ammonia and amines. For this study, typical amine molecules commonly used for industrial purposes (*e.g*., ammonia, cadaverine, hydrazine hydrate, methylamine, diethylamine, triethylamine, 1-naphthylamine, and pyridine) were selected as representative target analytes. To test the efficiency of TGH^+^•PD for the selective detection of organic amines, measured amounts of various amine analytes were exposed to an aqueous suspension of TGH^+^•PD and the resulting fluorescence response was recorded. When the suspension was exposed to increasing concentrations (0 M to 2.3 × 10^−6^ M) of ammonia, TGH^+^•PD exhibited a rapid luminescent response. The broad emission band of TGH^+^•PD, centered at ~553 nm, exhibited a 20 nm blue shift to a yellow-colored emission centered at ~533 nm (Fig. 3a). Thus, in response to the addition of NH_3_, a progressive increase in luminescence intensity was observed with a measured quantum yield of 5–7% (method section). This change in emission efficiency allowed for very efficient detection of ammonia, with a calculated detection limit of 1.2 × 10^−7^ M. This ultra-low limit of detection (LoD) value (Fig. 3b) outperformed or was comparable to most previously known chemosensors (Supplementary Table 1) for ammonia detection^6,12,68–70^. The use of other organic amines (cadaverine, hydrazine hydrate, methylamine, diethylamine, triethylamine, 1-naphthylamine, and pyridine) as analytes resulted in similar luminescence changes (Supplementary Figs. 18–24). Small molecules such as hydrazine hydrate, methylamine, and cadaverine showed similar interaction to ammonia. However, diffusion is limited for larger amine molecules such as trimethylamine, diethylamine, and 1-naphthylamine, producing a minimal response. Moreover, TGH^+^•PD contained a limited void space that can be accessible by the guest molecules as demonstrated by its low surface area of 16 m^2^ g^−1^. This low porosity of the COF is a result of the short length of the constituent linkers and the presence of counterions within the pore channels. This results in restriction of the entry of non-interacting guest molecules into the pore channels and therefore low N_2_ uptake was observed. However, it is noteworthy that the COF pores contained a helical water channel, which suggested that analytes similar in size such as ammonia can enter the pores and interfere with the ICT process. Therefore, it is possible that analyte molecules interact with the sensor from both the outer surface and the pore surface, depending on their size and interaction. A similar observation was reported previously^71^, where low porosity restricted the diffusion of analytes into the pores and effective sensing by surface interaction was observed.

In order to expand the applications of our COF material, we also performed sensing in the solid state, whereby fluorescence enhancement was observed for both powder and solution dispersions. To verify the luminescence properties in the powder, we activated the as-synthesized materials for 8 h to remove the solvent molecules from within the COF network. The activated COF material showed very weak emission in the solid state (Supplementary Fig. 25) with a maxima at 600 nm (*λ*_ex_ = 375 nm). Upon exposure to ammonia, the material showed fluorescence enhancement that can be visually observed under a UV lamp (Supplementary Fig. 25). We have also performed an additional vapor phase sensing study (Supplementary Figs. 26–27). Here, the dispersion of TGH^+^•PD was exposed to saturated analyte vapors in a closed state for 10 s at 25 °C and the emission property was measured. As expected, the fluorescence enhancement, in this case, is strongly dependent on the vapor pressure, since it determines the analyte concentration in the vapor phase. Interestingly, more than threefold enhancement was observed upon exposure to ammonia vapor, again demonstrating the capability of TGH^+^•PD as a luminescence sensor for the detection of ammonia in the vapor phase (Supplementary Fig. 26).

The difference in the luminescence responses could be related to either their bulkiness (triethylamine and diethylamine) and/or their high p*K*_b_ (pyridine and 1-naphthylamine), effectively limiting their interaction with the guanidinium units. The selectivity of TGH^+^•PD (Fig. 3c) towards ammonia and aliphatic primary amines is an important parameter for sensor development. We also tested the sensing capability of TGH^+^•PD towards other non-amine-based organic solvents, including benzene, toluene, nitrobenzene, and methanol (Supplementary Figs. 28–31). The fluorescence intensity of TGH^+^•PD changed minimally with these organic solvents. After exposure to ammonia, the original fluorescence intensity of TGH^+^•PD could be restored by adding equivalent amounts of HCl, and the material was tested for five consecutive cycles (for ammonia) without any significant loss in sensitivity, clearly indicating the durability of TGH^+^•PD (Fig. 3d) as a sensor. A similar observation was also made for other amines (Supplementary Fig. 32). The durability of the COF was further confirmed by PXRD analysis after COF regeneration, where the crystallinity of the framework remained unchanged after several cycles of acid/base treatment (Supplementary Fig. 33).

The addition of the amine analytes in water leads to an increase in pH up to a value of pH = 13. To verify the applicability of TGH^+^•PD as a sensing material, we tested its chemical stability by monitoring the PXRD patterns (Supplementary Fig. 34) and microscopic morphology (HRTEM, Supplementary Fig. 35) under different pH conditions. In addition to the alkaline pH range, we also checked their stability in the acidic pH range. The morphology and PXRD pattern remained unchanged under different pH conditions (Supplementary Fig. 34), and clearly indicated the robustness of the COF materials. The HRTEM images acquired for the materials at different pH values also showed a similar morphology with no signs of decomposition. Therefore, these experiments conclusively demonstrated the structural integrity of TGH^+^•PD under different pH conditions. TGH^+^• PD COF is very pH sensitive, the activated COF powder immediately turns dark red when exposed to an acid, orange when exposed to water, and greenish-yellow when soaked in a base for a few seconds (Supplementary Fig. 36a). This interesting property of optical response to pH changes was monitored by UV–Vis spectroscopy (Supplementary Fig. 36b). In all cases, a bathochromic red shift was observed with the increase in pH. To test the effect of pH on photophysical properties, we recorded the fluorescence emission spectra in acidic (pH = 2) and basic (pH = 13) environments for TGH^+^•PD (Supplementary Fig. 37). The fluorescence intensity of TGH^+^•PD is pH-dependent, and the intensity increased with increasing pH from 2 to 13 (Supplementary Fig. 37a, b). At basic pH, a significant enhancement of the emission with a blue shift (20 nm) was observed, which could be attributed to delocalization of electrons throughout the network by negatively charged N atoms (Supplementary Fig. 37c). A similar observation has also been reported previously^72^. To check the structural changes of iCOF at different pH values, we also recorded the ^13^C solid-state CP/MAS NMR spectra (Supplementary Fig. 38). It was clearly seen from the NMR spectra that the signal corresponding to the –C=N group of the imine and phenanthroline moieties of TGH^+^•PD COF experienced an obvious shift to higher fields at pH = 2. These results indicated that at acidic pH, protonation occurs at the imine sites within the COF network. On the other hand, at pH = 13, there is a noticeable downfield shift of the guanidinium carbon atom which indicated that deprotonation of the guanidinium moiety in basic medium had occurred. Solid-state NMR also revealed that the imine group of the COF network remained intact in both extremely acidic and basic conditions, which further confirmed their chemical stability.

The change in the emission spectra upon addition of ammonia suggests that the NH_3_ molecules trigger the deprotonation of TGH^+^•PD at the guanidinium sites and block the ICT process from phenanthroline to the guanidinium group. Zeta potential analysis of TGH^+^•PD revealed a negative surface potential of –12 mV after addition of NH_3_, compared with +14.5 mV before addition. This indicates that proton abstraction from TGH^+^•PD occurred upon addition of NH_3_. In the deprotonated state, the delocalization of electrons throughout the network is enhanced due to the negative charge of the nitrogen atom. This higher electron density is likely the cause of the enhanced emission intensity of TGH^+^•PD. This phenomenon of enhanced electron delocalization was explained by Mandal et al., using amide-hydrazide functionalized COFs, who observed a similar enhancement of fluorescence in the deprotonated form of the network^72^. The deprotonation process of TGH^+^•PD after ammonia treatment was also confirmed by solid-state ^13^C CP/MAS NMR (Supplementary Fig. 39). The CP/MAS analysis revealed that the TGH^+^•PD sample treated with ammonia exhibited a significant chemical shift of 1.7 ppm observed for the high-frequency ^13^C signals of the carbon atom of the guanidinium units. The other peaks of TGH^+^•PD remained unchanged indicating the chemical stability of the COF under basic conditions. Two-dimensional ^1^H-^13^C HETCOR (HETero-nuclear CORrelation, Supplementary Fig. 40) experiments were recorded for the TGH^+^•PD samples before and after treatment with ammonia. Supplementary Fig. 40 shows the superimposed two-dimensional ^1^H-^13^C HETCOR spectra of the TGH^+^•PD before (black contour) and after (red contour) treatment with ammonia. The spectra obtained from both samples were almost identical and showed mainly ^1^H-^13^C correlation peaks from the aromatic and guanidinium carbons with the aromatic protons. However, chemical differences were observed in the ^1^H-^13^C correlation peaks from the high-frequency guanidinium carbon atom. In the case of TGH^+^•PD treated with ammonia (red contours), the ^13^C chemical shift of the guanidinium carbon atom is shifted by 1.7 ppm to a higher frequency and the ^1^H chemical shift is shifted by 0.4 ppm to a lower frequency. Taken together, these results indicate that TGH^+^•PD can be used for the selective light-up detection of ammonia and aliphatic primary amines.

To some extent, similar results were also observed when the TGH^+^•PD COF was treated with a strong base such as NaOH (Supplementary Fig. 41a). However, even after adding a higher concentration of NaOH solution (10^−2 ^M), the emission enhancement observed was 1.2-fold lower than that of NH_3_. Therefore, the maximum dynamic range for fluorescence enhancement (3-fold) could be achieved only by the addition of NH_3_. To check the selectivity for NH_3_ (aq) in the presence of NaOH (aq), TGH^+^•PD COF was exposed to NaOH (aq) followed by NH_3_ (aq) (Supplementary Fig. 41b). Interestingly, following the addition of NH_3_ (aq), the fluorescence intensity of TGH^+^•PD COF was enhanced 3-fold. We also performed sensing studies with TGH^+^•PD-anh which showed similar luminescence changes upon exposure to ammonia, cadaverine, hydrazine hydrate, and methylamine (Supplementary Fig. 42, Supplementary Table 2).

### Solid-state thermochromism and role of water in sensing applications

**Fig. 4:**
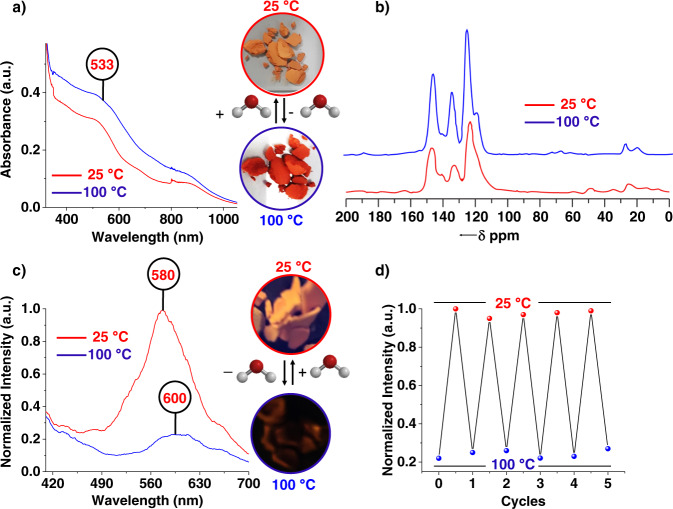
Solid-state thermochromic properties of TGH^+^•PD COF material. **a** UV–Vis–NIR spectra of unactivated TGH^+^•PD and its change after heating to 100 °C, inset showing thermochromic behavior of TGH^+^•PD with reversible color change. **b** ^13^C-CP/MAS NMR spectra of unactivated TGH^+^•PD and after heating to 100 °C [*δ* = chemical Shift]. **c** Emission spectra (*λ*_ex_ = 375 nm) of unactivated TGH^+^•PD (red line) and after heating to 100 °C (blue line). **d** Reversible change in fluorescence intensity of TGH^+^•PD upon alternate heating and cooling cycles (*λ*_ex_ = 375 nm).

As TGH^+^•PD COF contains a helical water assembly throughout the network, we monitored the photophysical behavior of COF samples with and without water molecules. Water molecules play a crucial role in the optical and photoluminescent properties of TGH^+^•PD. To investigate the role of water, UV–Vis spectroscopy and photoluminescence studies were performed in the solid state under completely dry conditions (Fig. 4). When the TGH^+^•PD was heated to 100 °C, the COF material showed a drastic and clearly visible color change from orange to deep red (inset Fig. 4a). This color change was confirmed by solid-state UV–Vis–NIR spectrophotometry, which revealed a distinct change in the intensity of the π→π* CT absorption (*λ*_max_ = 533 nm) band with increasing temperature (Fig. 4a). The color change was a completely reversible process when the heat is removed, with the original color being restored upon rehydration. To verify the structural integrity, the solid-state ^13^C CP/MAS spectrum was recorded for the activated TGH^+^•PD sample and no spectral changes could be detected (Fig. 4b). The absence of any spectral changes upon thermal treatment was further evidence of the outstanding stability of the material. To understand the mechanism for the color change at high temperatures, we recorded the PXRD analysis at 100 °C (Supplementary Fig. 13). At the thermochromic transition temperature (100 °C), the positions of the diffraction peaks in the wide-angle region changed significantly along with the color change, indicating that the color change originates from a structural change in the crystal packing of the TGH^+^•PD COF upon the removal of entrapped water molecules within the network. This type of observation has also been reported in other thermochromic materials^73–75^. To gain deeper insight into the role of water in the material’s photophysical properties, photoluminescence studies of the activated material were performed in the solid state (Fig. 4c). The activated material showed very weak emission at 600 nm (at *λ*_ex_ = 375 nm). Upon exposure to humidity, the material showed a fivefold increase in fluorescence intensity with an emission maximum at 580 nm (at *λ*_ex_ = 375 nm) accompanied by a blue shift of about 20 nm. This enhancement of emission intensity with blue shift of emission maxima can be seen with the naked eye (inset, Fig. 4c). When the temperature was increased to 100 °C, the emission is significantly attenuated, showing the reversible luminescent thermochromic behavior of the compound. These temperature-induced fluorescence “on–off” switching experiments were performed for up to five consecutive cycles (Fig. 4d) without any decrease in emission intensity. The enhanced fluorescence intensity is expected in the hydrated state due to the formation of intermolecular hydrogen bonding throughout the extended network. These hydrogen bonds increase the material’s molecular rigidity causing the intramolecular motions to be restricted, a process which is associated with a decrease in non-radiative transitions, leading to enhanced fluorescence. Similarly, enhancement of fluorescence by hydrogen bonding using water is also reported in the literature^76,77^.

To verify the role of water in sensing, we performed solid-state sensing for ammonia and other amines in both dehydrated and hydrated COF samples (Supplementary Fig. 43). Interestingly, TGH^+^•PD in the dehydrated state showed fluorescent “turn on” response to ammonia vapor (Supplementary Fig. 43), with a significant increase in emission intensity (*λ*_max_ = 571 and *λ*_ex_ = 375 nm) and a blue shift of emission maxima of ~30 nm. Hydrated COF also showed a similar response to ammonia vapor, but the emission intensity of the COF in the hydrated state is more dominant compared to the dehydrated COF samples. Exposure of COFs to other amines showed no response in the solid state because of the lower vapor pressure of the amines. This observation highlights the remarkable selectivity for NH_3_ detection in the solid state.

### Monitoring of meat freshness

**Fig. 5:**
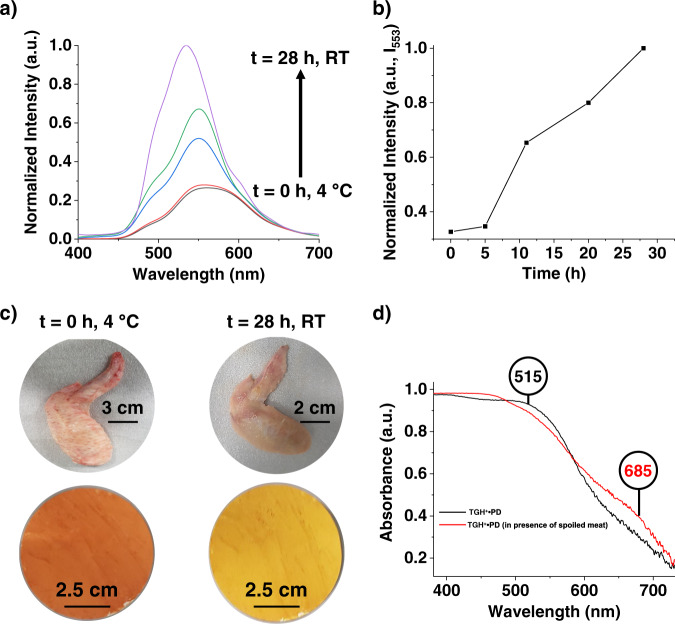
Monitoring the meat freshness visually by TGH^+^•PD COF. **a** Emission response of a TGH^+^•PD dispersion in water at different time intervals in the presence of a chicken wing kept at room temperature (RT). **b** Fluorescence intensity values were measured over different exposure times at room temperature. **c** Photographs of filter paper coated with TGH^+^•PD powder when exposed to fresh (left) and spoiled (right) chicken meat. **d** Overlaid solid-state UV–Vis spectra of a TGH^+^•PD-coated test strip, recorded at room temperature in the presence of fresh and spoiled chicken meat.

Given that ammonia and biogenic amines such as cadaverine are released during the decomposition of meat proteins, their detection is essential for monitoring and evaluating the quality of meat products during transportation and storage. To address this need, we tested the ability of TGH^+^•PD for in situ monitoring of meat spoilage. In a typical experiment, a piece of fresh chicken meat was first stored for 2 days at 4 °C in a sealed beaker to simulate typical storage conditions; along with a UV quartz cuvette containing an aqueous suspension of TGH^+^•PD. Over the next two days, an insignificant change in fluorescence intensity was observed (Supplementary Fig. 44), due to minimal release of ammonia and biogenic amines at 4 °C. However, once the sealed beaker was removed from the refrigerator and stored at room temperature, the recorded fluorescence spectra showed a significant increase in luminescence intensity (Fig. 5a, b) over time. This is a clear indication that TGH^+^•PD is capable of detecting the amines released from spoiled meat and can be used as a freshness control for such meat products.

To further demonstrate the practical utility of TGH^+^•PD, we studied the applicability of the chemosensor in the solid state. Since TGH^+^•PD COF is readily dispersible in water, we prepared a paper strip by applying its aqueous dispersion to a filter paper. The test strip was then suspended in a container over a piece of chicken meat (Supplementary Fig. 45) and underwent a distinct color change from orange to yellow-green (Fig. 5c) as the meat began to rot and emit amine vapors. The observed color change was characterized by solid-state UV–Vis spectroscopy (Fig. 5d). When the COF-coated paper was exposed to spoiled meat, the original absorption peak at 515 nm decreased and a new band appeared at 668 nm, which was attributed to the deprotonation of the COF with amines released when meat spoiled. After a control experiment, the solid-state UV-Vis spectrum of TGH^+^•PD COF in the presence of ammonia showed the same changes (Supplementary Fig. 46, Supplementary Movie 1). The original color of the COF-coated filter paper could be easily regenerated by keeping the filter paper in fresh air and reusing the regenerated paper. This approach provides a portable, rapid, and inexpensive detection method for determining meat freshness^78^, unlike other detection methods that rely mainly on complex and expensive techniques such as chromatography^79^, electrochemistry^80^, capillary electrophoresis^81^, and chemiluminescence^6^.

## Discussion

In summary, an efficient “turn-on” chemosensor for the detection of ammonia and biogenic amines has been developed by exploiting the emission property of TGH^+^•PD iCOF. The iCOF exhibits ICT-based emission at 553 nm. We have successfully investigated the selective “turn-on” sensing properties of TGH^+^•PD for the detection of ammonia and primary amines by inhibiting the ICT phenomenon through deprotonation of the guanidinium groups of the COF. The immediate increase in emission intensity highlights the usefulness of TGH^+^•PD as a chemosensor for ammonia with a very low limit of detection (LoD ∼ 2.04 ppb). Moreover, TGH^+^•PD has also been shown to be efficient for monitoring the freshness of meat products that release amine vapors upon spoilage. The experimental results clearly demonstrate that strategically designed COFs can be used as a portable, cost-effective, and highly sensitive technology suitable for rapid detection of amine vapors and evaluation of meat product freshness.

## Methods

All reagents and starting materials were purchased from Sigma-Aldrich and used without further purification. Synthesis of 2,9-diformyl 1,10-phenanthroline (PD) and triamino guanidium hydrochloride salt (TGH•Cl) was synthesized as previously reported literature^46,82^. Column chromatography was performed on silica gel 60F (Merck 9385, 0.040–0.063 mm). All amine analytes were purchased from Sigma Aldrich and used without further purification, purity of all analytes is ammonium hydroxide (28.0–30.0%), methyl amine (2 M in THF), hydrazine hydrate (80%), pyridine (99%), 1-naphthylamine (99%), cadaverine (95%), diethylamine (99.5%), and triethylamine (99.5%). Solution state nuclear magnetic resonance (NMR) spectra were recorded at 25 °C on a Bruker Avance III spectrometer, with working frequencies of 500 MHz for ^1^H, and 125.0 MHz for ^13^C nuclei. All chemical shifts are reported in ppm relative to the signals corresponding to the residual non-deuterated solvents (CDCl_3_ = 7.26 ppm)^83,84^. All ^13^C NMR spectra were recorded with the simultaneous decoupling of proton nuclei. Coupling constant values (*J*) are given in hertz (Hz). The multiplicity of the proton spectrum is abbreviated in the following way: s (singlet), d (doublet), dd (doublet of doublets), t (triplet), q (quartet), qt (quintet), sx (sextet), m (multiplet) and a wide signal is preceded by br (broad). All solid-state NMR experiments were carried out on a Bruker Avance-HD 600 MHz spectrometer operating at a static field of 14.1 T, resonating at 150.0 MHz for ^13^C, using a 4.0 mm double resonance MAS probe. High-resolution mass spectrometry analyses were performed using an Agilent 6540 UHA Accurate Mass Q-TOF/LC-MS-spectrometer in the positive mode and an acetonitrile/water used a gradient in C18 column. Fourier transform infrared (FTIR) studies were carried out on the Agilent 670-IR spectrometer. Thermogravimetric analysis (TGA) was performed on TA SDT Q600. SEM images were obtained from FEI Quanta 450FEG. The topography of the TGH^+^•PD was analyzed by dynamic atomic force microscopy (5500 Atomic Force Microscope; Keysight Technologies Inc., Santa Rosa, CA). We acquired topography, phase and amplitude scans simultaneously. Silicon cantilevers (Nanosensors^TM^, Neuchatel, Switzerland) with resonant frequencies of 250–300 kHz and force constants of 100–130 N m^−1^ were used. The set point value was kept at 2.5 V. AFM scans were collected at 1024 points/lines with scan speed of 0.20 at fixed scan angle of 0°. Scan artifacts were minimized by acquiring a typical scan at an angle of 90° under identical image acquisition parameters. We used GwyddionTM free soſtware (version 2.47), SPM data visualization and analysis tool for post-processing the AFM scans. Size and morphology of the COF material were determined with a TEM (FEI-Titan 300) microscope. Samples were prepared on a carbon-coated copper grid. A drop of dispersed TGH^+^•PD network was spotted on the grid and allowed to dry overnight. Surface area measurements were conducted on a Micromeritics 3Flex gas sorption analyzer. Samples (20–50 mg) were degassed at 85 °C for 24 h and then backfilled with N_2_. Adsorption isotherms were generated by incremental exposure to ultrahigh-purity nitrogen up to 1 atm in a liquid nitrogen bath, and surface area was determined using BET adsorption models included in the instrument software (Micromeritics ASAP 2020 V4.00). PXRD measurements were carried out using the PANalytical X’Pert PRO MP X-ray diffractometer consisting of a focusing elliptical mirror and a fast-high resolution detector (PIXCEL) with the radiation wavelength of 0.15418 nm. Circular dichroism spectra were measured at room temperature using a Chirascan CD Spectrometer from Applied Photophysics. Fluorescence quantum yield was calculated by using the known equation^85^. A drop of dispersed polymeric network was spotted on the grid and allowed to dry overnight. UV–Vis studies were carried out on the Cary 5000 UV–Vis–NIR spectrophotometer. Emission spectra in water at room temperature were recorded on a Perkin Elmer LS55 Fluorescence Spectrometer. Dynamic light scattering measurements were performed on a Malvern Zeta sizer NanoSeries to obtain the size and zeta-potential of the nanoparticles.

### 1. Synthesis of 2,9-diformyl 1, 10-phenanthroline (PD)


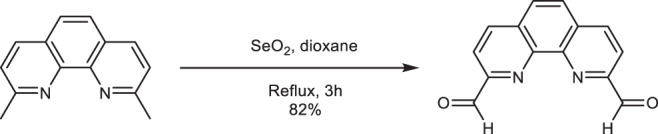


The **PD** linker was synthesized by following the literature with slight modification^86^. 2,9-dimethyl-1,10-phenanthroline (1.50 g, 6.9 mmol) and selenium dioxide (3.25 g, 33.8 mmol) were refluxed for 3 h with 100 ml of 1,4-dioxane in presence of 4% water. After the completion of the reaction, the mixture was filtered in hot condition through a celite bed and the product was precipitated from the solution by cooling to 0 °C. The precipitate was collected by filtration and the PD linker was further purified by recrystallization from hot dioxane. Yield: 1.40 g, 82%; ^1^H NMR (500 MHz, DMSO-d_6_, 25 °C): δ 8.31 (s, 2H, Ar-*H*), 8.33 (d, 2H, *J* = 8.3 Hz, Ar-*H*), 8.81 (d, 2H, *J* = 6.2 Hz, Ar-*H*), 10.38 (s, 2H, Ar-C*H*O); ^13^C NMR (125 MHz, DMSO-d_6_, 25 °C): δ 120.7, 129.7, 131.8, 138.9, 145.9, 152.7, 194.3.

### 2. Synthesis of triamino guanidium hydrochloride salt (TGH•Cl)


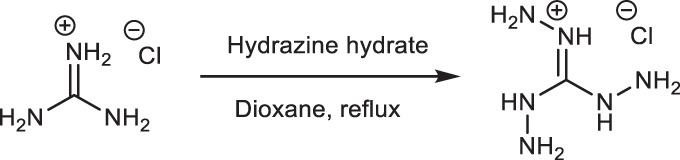


Triaminoguanidinium chloride (TGH•Cl) was synthesized according to the published procedure^46^. 1.91 g of guanidine hydrochloride was added to 10 mL of 1,4-dioxane under stirring condition. To it, 3.41 g of hydrazine hydrate was added; the mixture was refluxed for 2 h. Then cooled to room temperature, filtered and washed with 1,4-dioxane to remove excess hydrazine hydrate and finally dried to yield TGH•Cl (Yield: 97%).

## Supplementary information

Supplementary Information

Description of Additional Supplementary Files

Supplementary Movie 1

## Data Availability

Data discussed in this study are presented in the text and the Supplementary Information. Additional data can be obtained from the corresponding author upon reasonable request. Calculated structure of TGH^+^•PD in CIF format can be obtained from www.ccdc.cam.ac.uk/structures with identifier 2125606.
