# Supplementary material for: Fluorescence turn on amine detection in a cationic covalent organic framework
Source: Nat Commun. 2022 Jul 7;13:3904. doi: 10.1038/s41467-022-31393-2 (PMC9263141; doi:10.1038/s41467-022-31393-2)
Supplement: Supplementary file 1 — Supplementary Information [file 41467_2022_31393_MOESM1_ESM.pdf]

Supplementary Information

Fluorescence Turn on Amine Detection in a Cationic Covalent Organic Framework

Gobinda Das,[1] Bikash Garai,[1,2] Thirumurugan Prakasam,[1] Farah Benyettou,[1] Sabu Varghese,[3] Sudhir Kumar Sharma,[4] Felipe Gándara,[5] Renu Pasricha,[3] Maria Baias,[1] Ramesh Jagannathan,[4] Na'il Saleh,[6,7] Mourad Elhabiri,[8] Mark A. Olson,[9] and Ali Trabolsi*[1,2]

[1] Chemistry Program, New York University Abu Dhabi (NYUAD), Saadiyat Island, United Arab Emirates

[2] NYUAD Water Research Center, New York University Abu Dhabi (NYUAD), Saadiyat Island, United Arab Emirates

[3] CTP, New York University Abu Dhabi, Abu Dhabi, UAE

[4] Engineering Division, New York University Abu Dhabi (NYUAD), United Arab Emirates

[5] Materials Science Institute of Madrid – CSIC, Sor Juana Inés de la Cruz 3, 28049 Madrid, Spain

[6] Chemistry Department, College of Science, United Arab Emirates University, P.O. Box 15551, Al Ain, United Arab Emirates

[7] National Water and Energy center, United Arab Emirates University, P.O. Box 15551, Al Ain, United Arab Emirates.

[8] Université de Strasbourg, Université de Haute-Alsace, CNRS, LIMA, UMR 7042, Equipe Chimie Bioorganique et Médicinale, ECPM, 25 Rue Becquerel, 67000 Strasbourg, France

[9] Department of Physical and Environmental Sciences, Texas A&M University Corpus Christi, 6300 Ocean Dr., Corpus Christi, TX 78412 USA

Corresponding Authors

*** ali.trabolsi@nyu.edu**

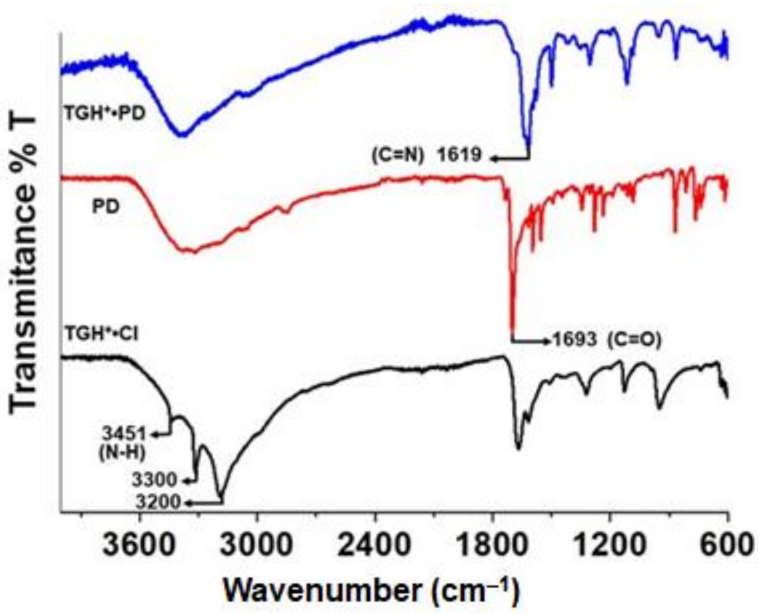

Supplementary Fig. 1. FTIR analysis of TGH$^+$•PD and its precursors. Stacked FTIR spectra of the as-synthesized TGH$^+$•PD (blue line), phenanthroline-2,9-dicarbaldehyde (PD, red line) and triamino guanidium hydrochloride salt (TGH$^+$•Cl, black line).

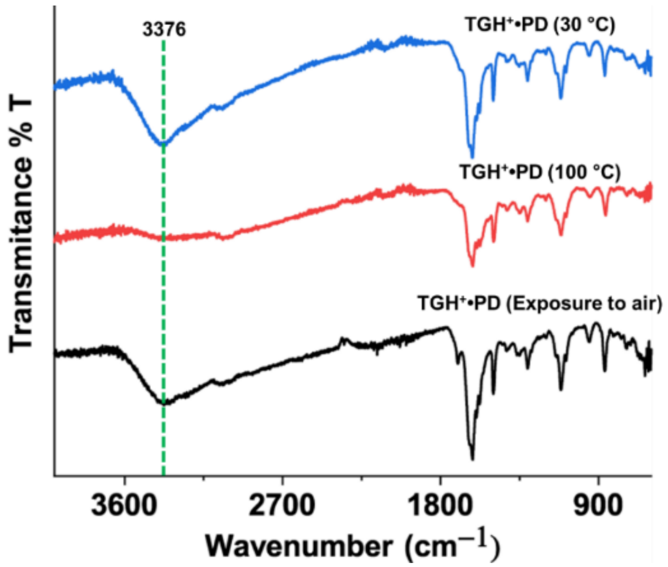

Supplementary Fig. 2. FTIR analysis of TGH$^+$•PD after regeneration. FTIR analysis of the as-synthesized TGH$^+$•PD (blue line), after heating at 100 °C in vacuum oven (red line) and after cooling down the sample to room temperature in open air (black line).

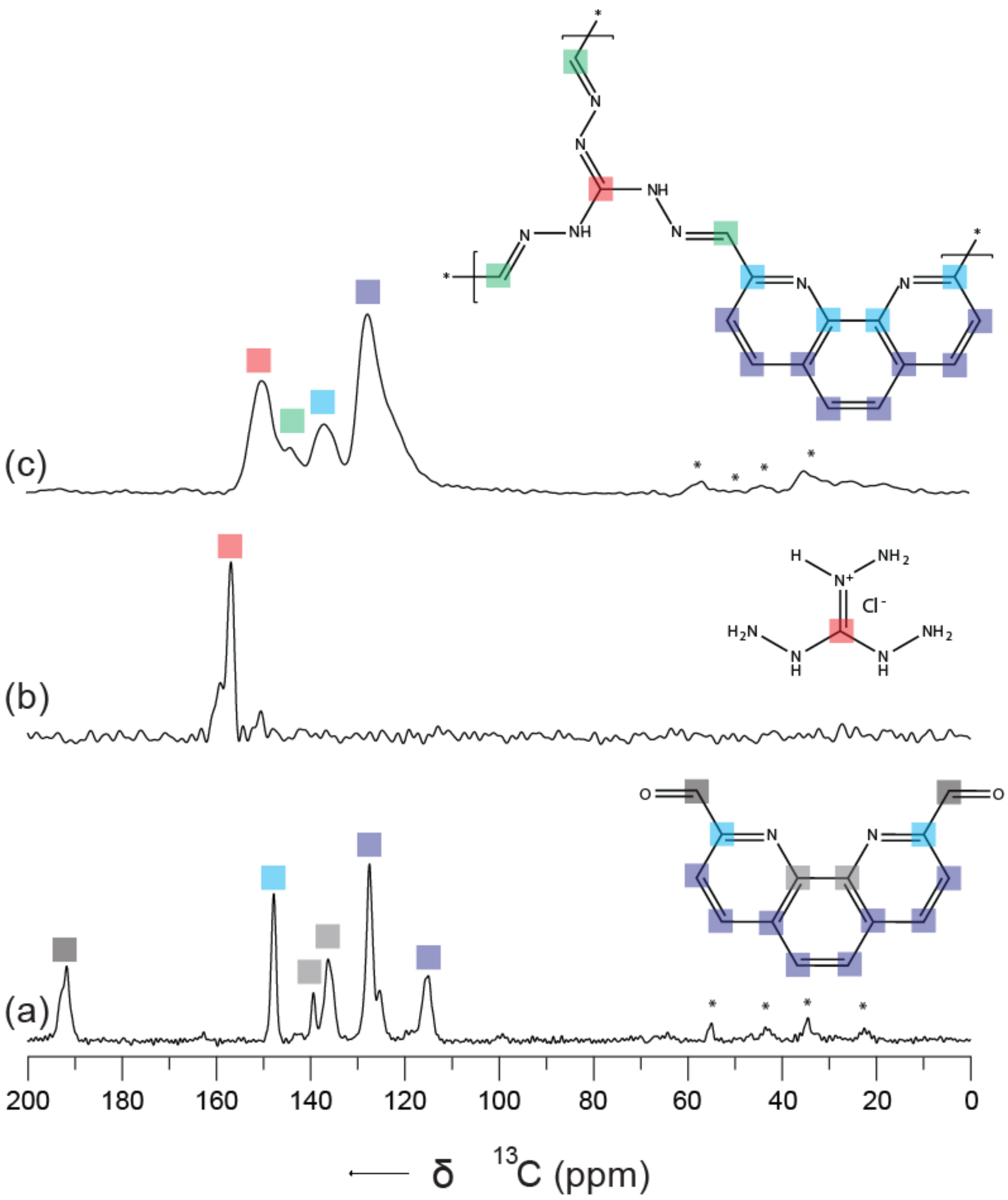

Supplementary Fig. 3. Solid state ^{13}C cross-polarization magic-angle spinning NMR spectral analysis. Stacked one-dimensional ^{13}C CP/MAS spectra of a) 1,10-Phenanthroline-2,9-dicarbaldehyde, b) TGH^{+}•Cl and c) TGH^{+}•PD. Spinning side bands are shown by asterisks.

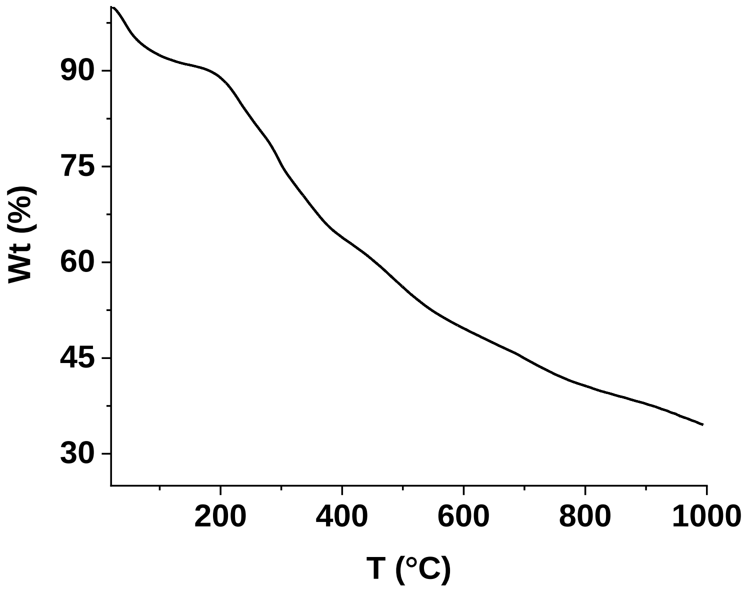

Supplementary Fig. 4. Thermogravimetric analysis (TGA) of TGH⁺•PD. TGA profile of TGH⁺•PD showing the thermal stability of the COF, the profile was recorded at a scan rate of 5 °C/min.

Water loss in the temperature range (25-100 °C) = 9.7%,

i.e. weight% of anhydrous unit = 90.3

Molecular weight of the asymmetric unit, $C_{44}H_{30}Cl_2N_{18}$ = 881.74

Each unit cell contains 2 asymmetric units, therefore, molecular weight of anhydrous COF unit cell = 1763.5

Thus, water content in unit cell = 1763.5 × 9.7/90.3 = 189.43 a.u.

i.e. 189.43/18 = 10.5 molecules per unit cell.

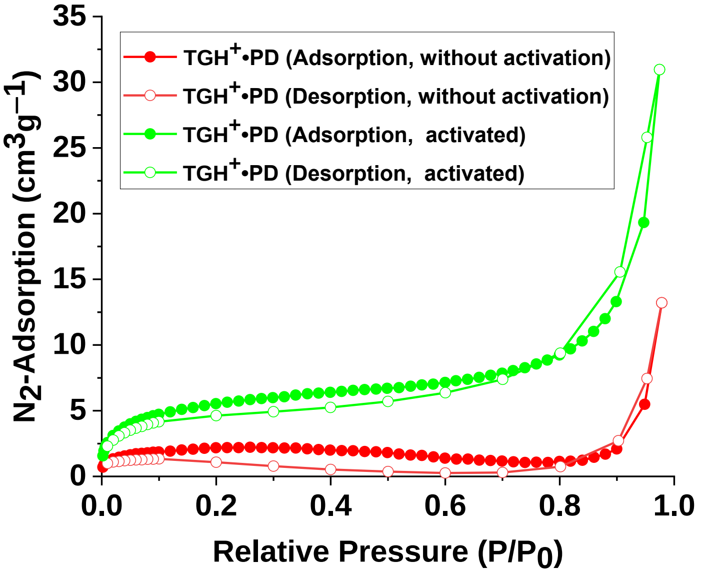

Supplementary Fig. 5. Porosity analysis of TGH⁺•PD. Comparison of the N_2 adsorption isotherms of the unactivated TGH⁺•PD COF (red) and the activated one (green). The activation of the sample was carried out at 80 °C for 12 hours under high vacuum.

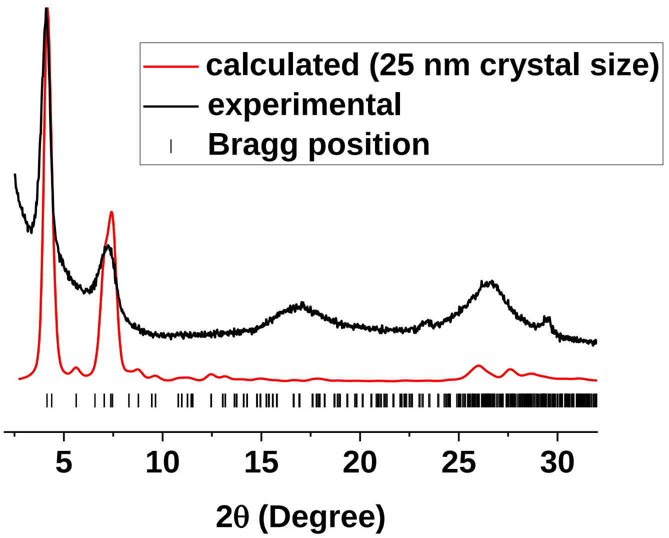

Supplementary Fig. 6. PXRD analysis of TGH⁺•PD. Comparison of experimental PXRD pattern and calculated from the proposed crystal structure including peak broadening by crystal size (25 nm).

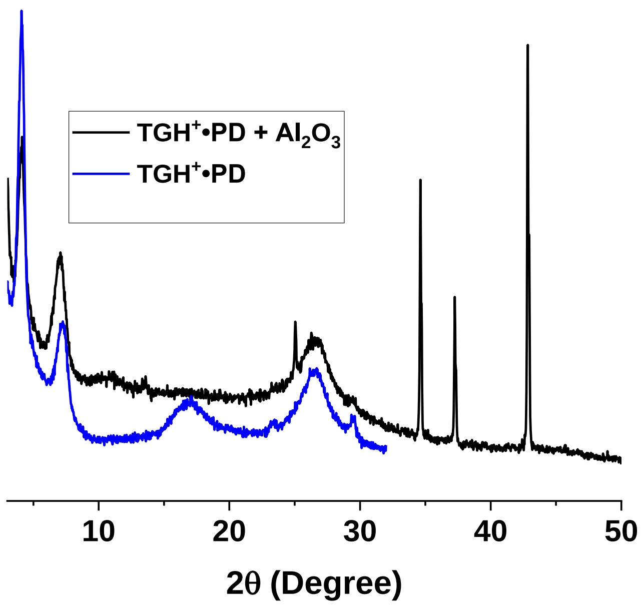

Supplementary Fig. 7. PXRD analysis of TGH$^+$•PD mixed with Al_2O_3. PXRD patterns of TGH$^+$•PD sample alone (blue), and mixed with Al_2O_3 (black). Note the disappearance of the broad feature in the 2θ = 15º-20º range, attributable to scattering by sample holder.

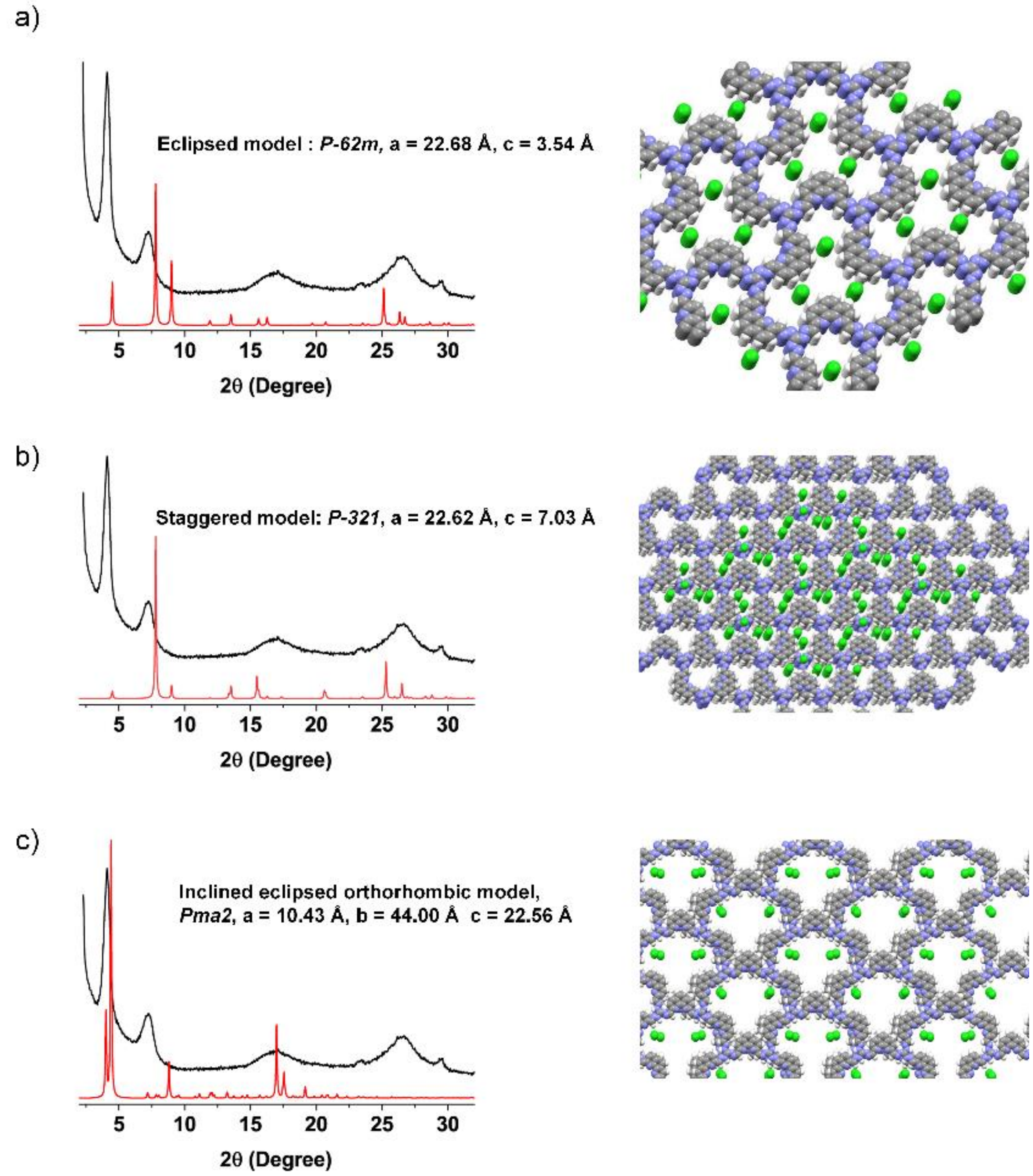

Supplementary Fig. 8. Simulated structure of TGH^{+}•PD COF in different packing models. Experimental PXRD patterns of TGH^{+}•PD COF compared with different probable stacking models [a) eclipsed model in *P*-62*m* space group, b) staggered model in *P*-321 space group, and c) inclined eclipsed orthorhombic model in *Pma*2 space group].

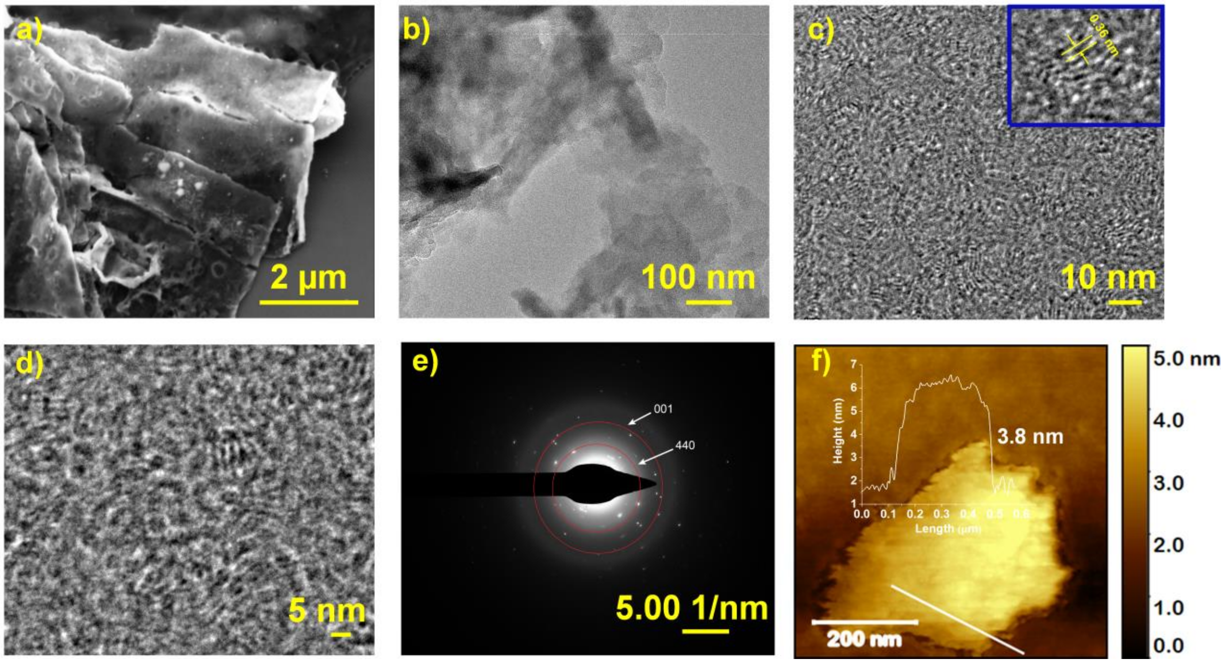

Supplementary Fig. 9. Detailed microscopic characterization after exfoliation. a) SEM, b-e) HRTEM, and f) AFM analysis of exfoliated TGH^{+}•PD COF material at different magnifications. Inset figure (c), indicates the *d*-spacing between the stacked layers and (f) height profile images.

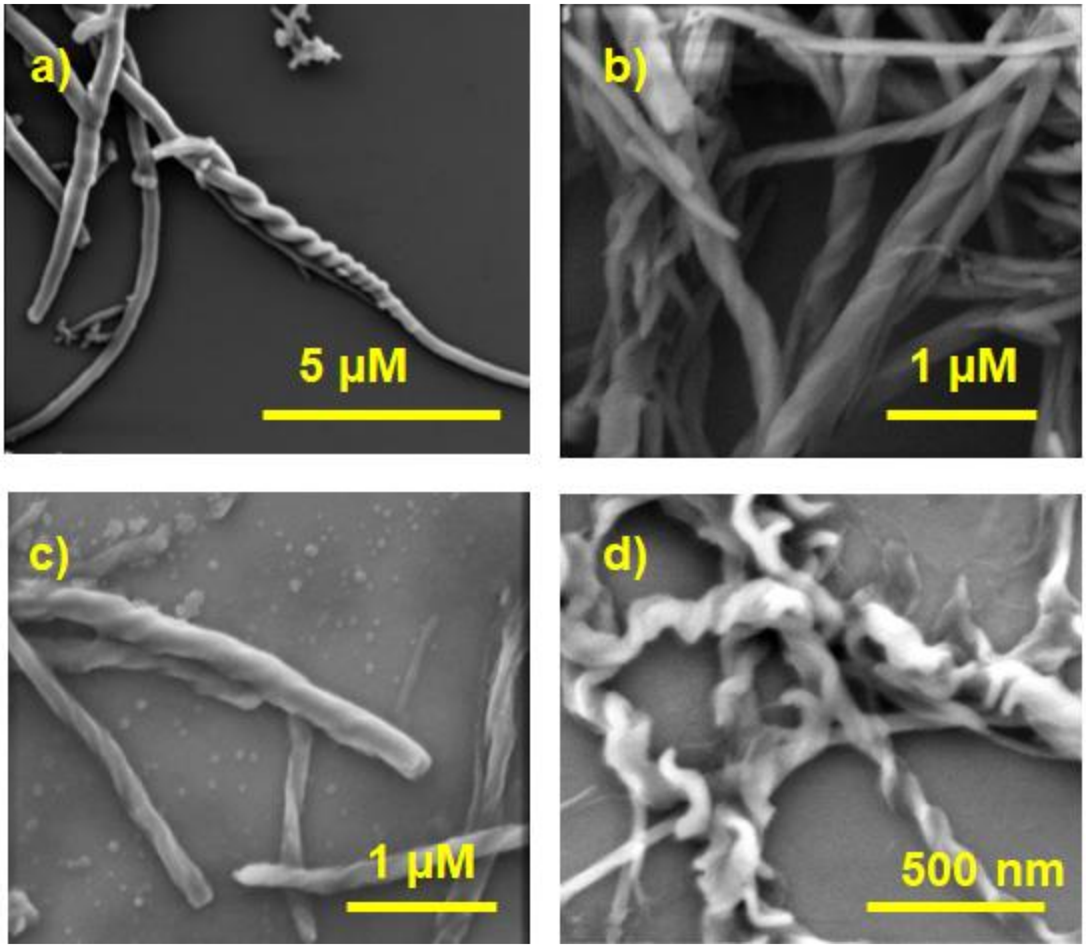

Supplementary Fig. 10. SEM analysis of TGH$^+$•PD. SEM (a-d) images of TGH$^+$•PD in different magnifications. SEM images showing both a) left and d) right-hand twisting features.

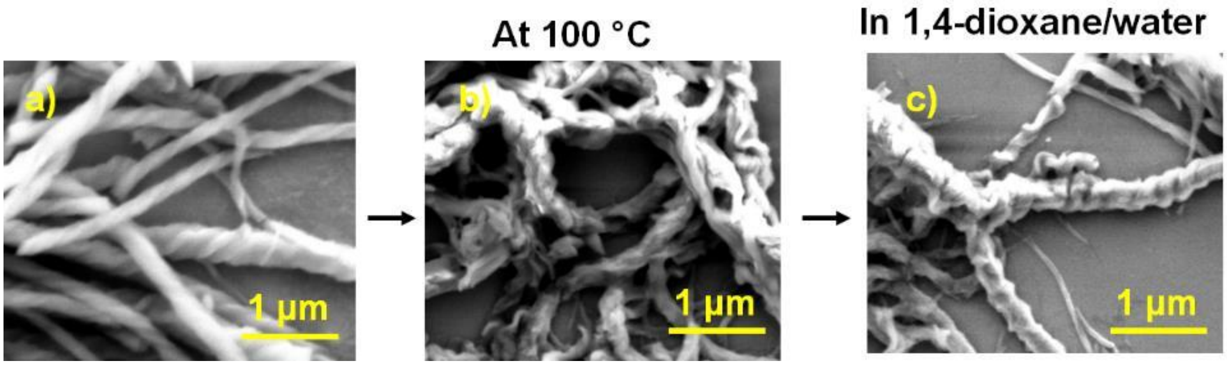

Supplementary Fig. 11. Effect of temperature on helical morphology. a) SEM images of the as-synthesized product; b) heating of the original helical fibers at 100 °C under vacuum for 12 h; c) regeneration of helical fiber after immersing the sample in dioxane + water system at room temperature.

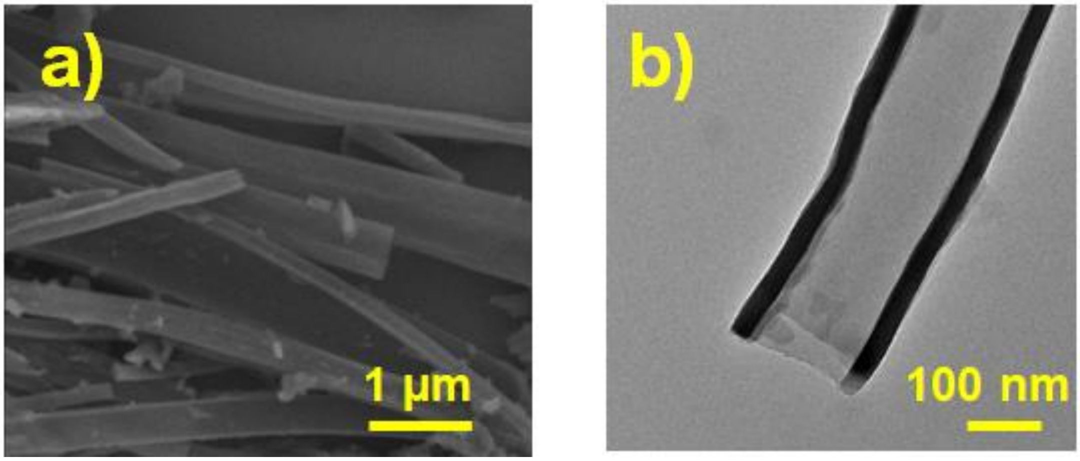

Supplementary Fig. 12. SEM analysis of TGH^{+}•PD synthesized in anhydrous 1,4-dioxane. a) SEM and b) TEM images of the product obtained from the solvothermal reaction in anhydrous 1,4-dixoane at 120 °C.

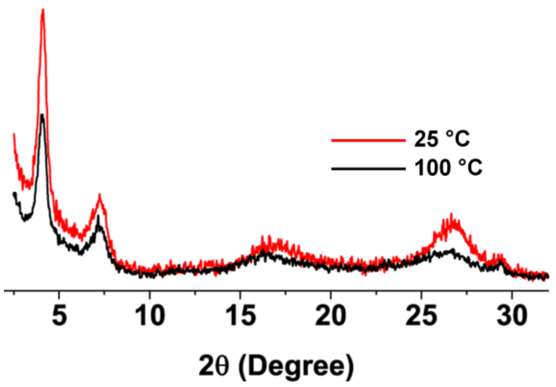

Supplementary Fig. 13. Variable temperature PXRD analysis of TGH^+•PD. Comparison of the PXRD patterns of the hydrated TGH^+•PD (red) and the activated (black). The reflection intensities, in particular at low angles, are significantly reduced in the activated state. The activation of the sample was carried out at 100 °C for 12 hours under high vacuum.

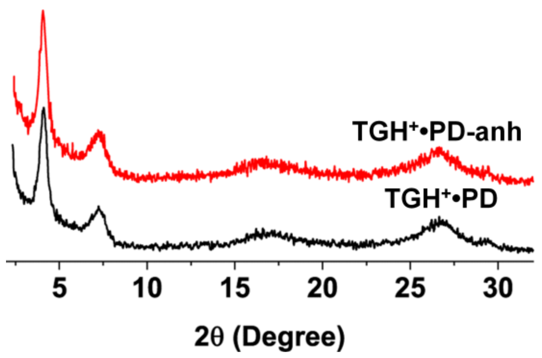

Supplementary Fig. 14. PXRD analysis of TGH^+•PD. in anhydrous 1,4-dioxane. Comparison of the PXRD patterns of TGH^+•PD (synthesized from ethanol/water, black pattern) and TGH^+•PD-anh (synthesized from anhydrous 1, 4-dioxane, red pattern).

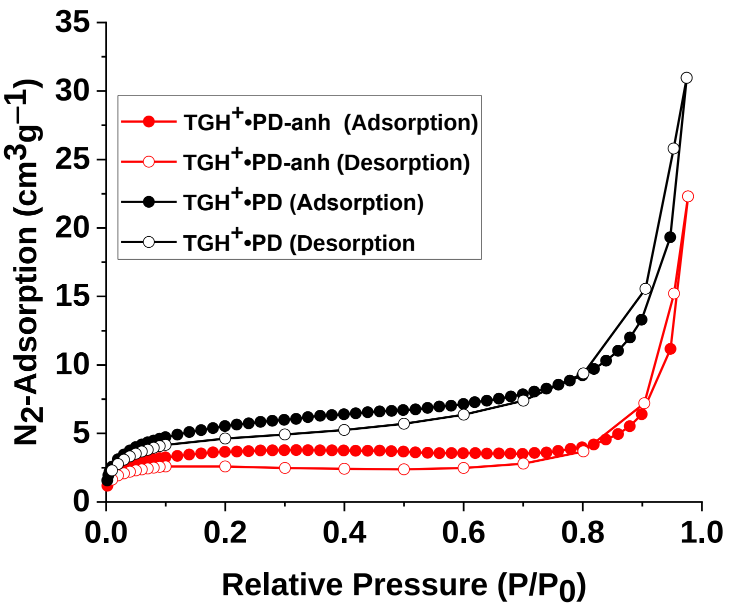

Supplementary Fig. 15. **Porosity analysis of TGH$^+$•PD. in anhydrous 1,4-dioxane.** Comparison of N_2 adsorption isotherm for TGH$^+$•PD (black) and TGH$^+$•PD-anh (red).

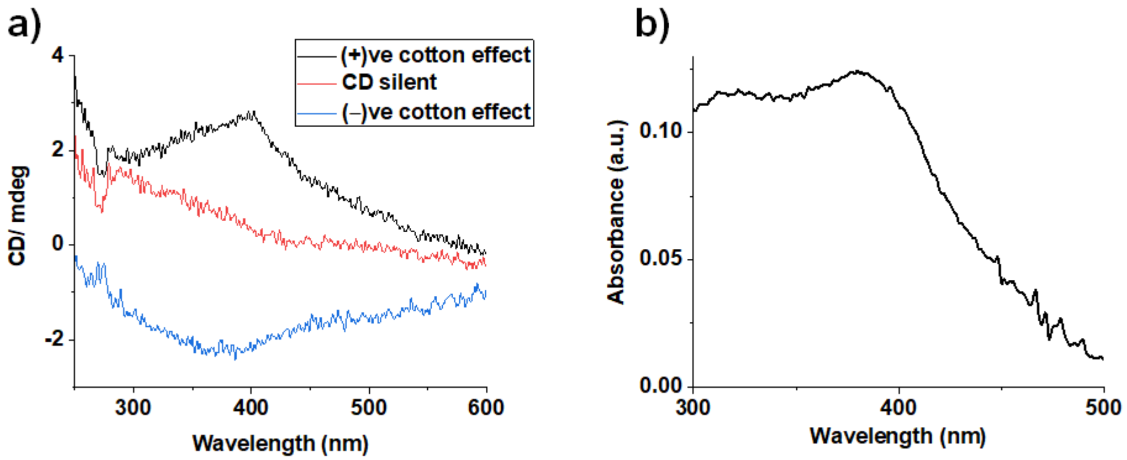

Supplementary Fig. 16. **Circular dichroism (CD) analysis**. a) Circular dichroism (CD) spectra of thin films prepared from the suspension of 5 mg TGH$^+$•PD in 1,4-dioxane/H_2O (1:0.6, v/v); b) UV−Vis absorption spectrum of TGH$^+$•PD. All the spectra were recorded at room temperature.

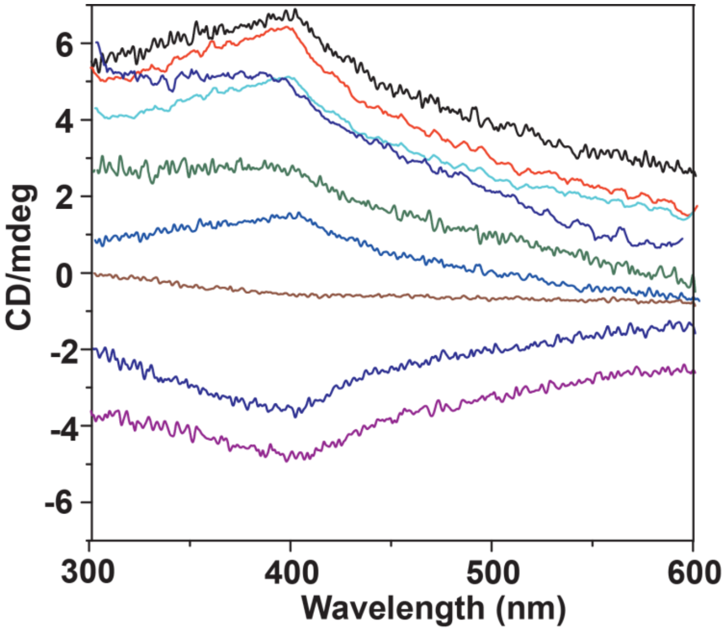

Supplementary Fig. 17. **Circular dichroism (CD) analysis**. CD spectra of different batches of TGH^{+}•PD thin films showing random cotton effect.

Supplementary Tab. 1. Comparison of amine-sensor materials reported in recent literatures

No	Materials	Detection of analytes	Media	Mode of Detection	LOD (ppm)	Applications
1	MOF, Zn_2(TCPE) and Mg(H_2DHBDC)[1]	NH_3 (g)	Solid state	Fluorescence "Turn on"	NA	NA
2	MOF, Zr-BTDB-fcu-MOF[2]	CH_3-NH_2 and Aniline	Water	Fluorescence "Turn on/off"	0.002-0.014	NA
3	MOF, Mg–NDI[3]	Hydrazine, Aniline	Solid state	Fluorescence "Turn off"	4.5	NA
4	Polymer[4]	NH_3 (aq)	Solid state	Fluorescence "Turn on"	5-2.5×10^4	Food spoilage detection
5	COF, TPE-Ph COF[5]	NH_3 (aq)	Toluene	Fluorescence "Turn off"	1	NA
6	MOF, [Zn(PA)(BPE)][6]	Ethylenediamine	Solid/DMSO	Fluorescence "Turn on"	9.9	NA
7	Porous polymers[7]	CH_3-NH_2 and Aniline	Water	Fluorescence "Turn on/off"	0.002-0.007	NA
8	Crosse Linked polymer[8]	NH_3 (g)	Solid state	Fluorescence "Turn on"	NA	NA
9	Dihydroquinoxaline (DQ) derivatives[9]	Primary amines	MeOH/Solid state	Fluorescence "Turn on"	0.69	Food spoilage detection

10	Pyrylium Salts[10]	NH_3 (g)	DCM	Fluoresce “Turn off”	NA	Food spoilage detection
11	Fluorescent carbon quantum dots[11]	NH_3 (g)	Solid state	Fluorescence “Turn on”	250–2.5 $\times 10^4$	Food spoilage detection
12	meso-Active-Ester-BODIPYs[12]	NH_3 (g)	Solid state	Fluorescence “Turn on”	NA	Food spoilage detection
13	MOF, EuMOF-FITC[13]	Biogenic amines	Solid state	Fluorescence “Turn on”	5-50	Food spoilage detection
14	MOF, (MR@EuMOFs))[14]	Histamine	Solid state	Fluorescence “Turn on”	0.011	Food spoilage detection
15	Polydiacetylene[15]	NH_3 (g)	Solid state	Colorimetric	100–1000	Food spoilage detection
16	This work	NH_3 (g) and Primary amines	Water/Solid state	Fluorescence “Turn on”, Colorimetric	0.002-0.004	Food spoilage detection

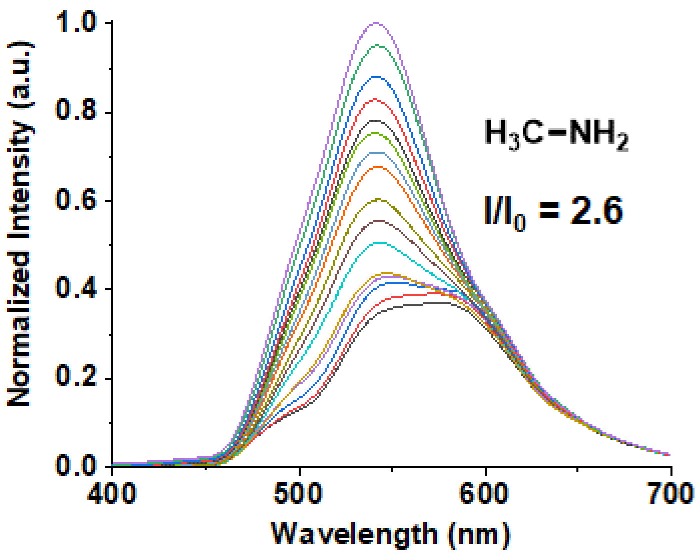

Supplementary Fig. 18. Luminescent spectra of TGH^+•PD in response to CH_3-NH_2. Changes observed on the emission spectrum of TGH^+•PD dispersed in water after exposure to methylamine (0-10^{-6} M). The emission spectra were recorded at room temperature (λ_{ex} = 365 nm).

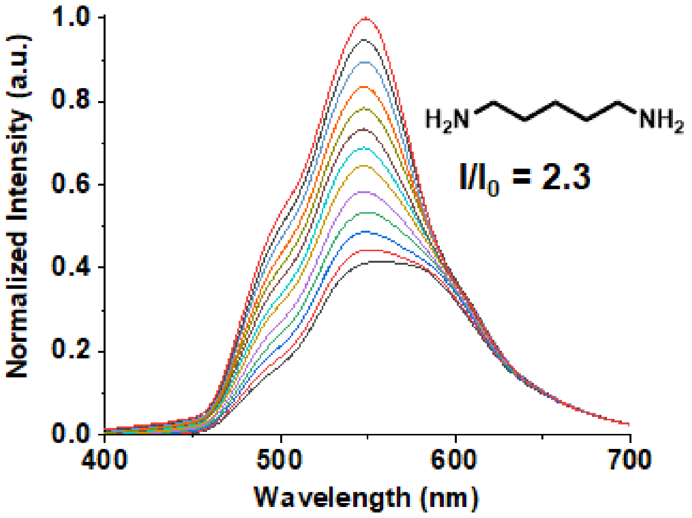

Supplementary Fig. 19. Luminescent spectra of TGH$^+$•PD in response to cadaverine
Changes observed on the emission spectrum of TGH$^+$•PD dispersed in water after exposure to cadaverine (0-10^{-6} M). The emission spectra were recorded at room temperature (λ_{ex} = 365 nm).

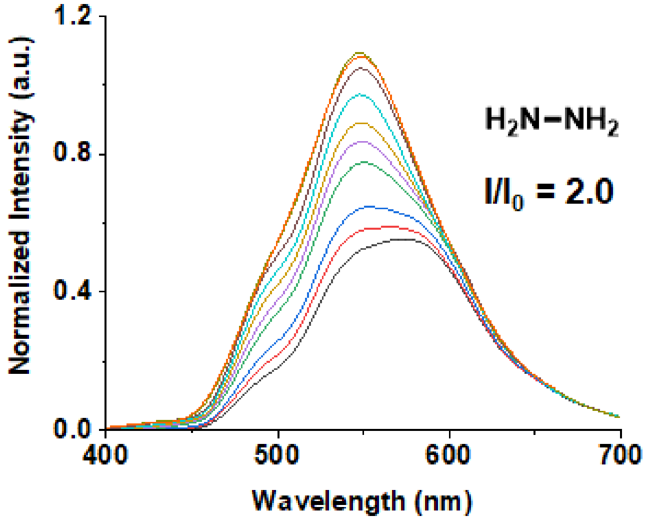

Supplementary Fig. 20. Luminescent spectra of TGH$^+$•PD in response to hydrazine hydrate
Changes observed on the emission spectrum of TGH$^+$•PD dispersed in water after exposure to hydrazine hydrate (0-10^{-6} M). The emission spectra were recorded at room temperature (λ_{ex} = 365 nm).

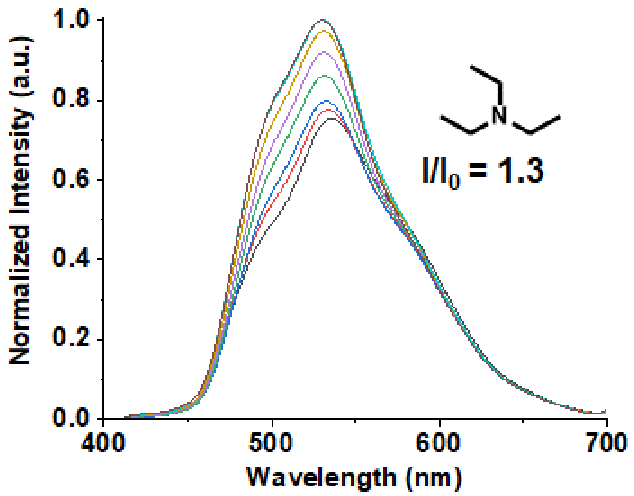

Supplementary Fig. 21. Luminescent spectra of TGH$^+$•PD in response to triethylamine Changes observed on the emission spectrum of TGH$^+$•PD dispersed in water after exposure to trimethylamine (0-10^{-6} M). The emission spectra were recorded at room temperature (λ_{ex} = 365 nm).

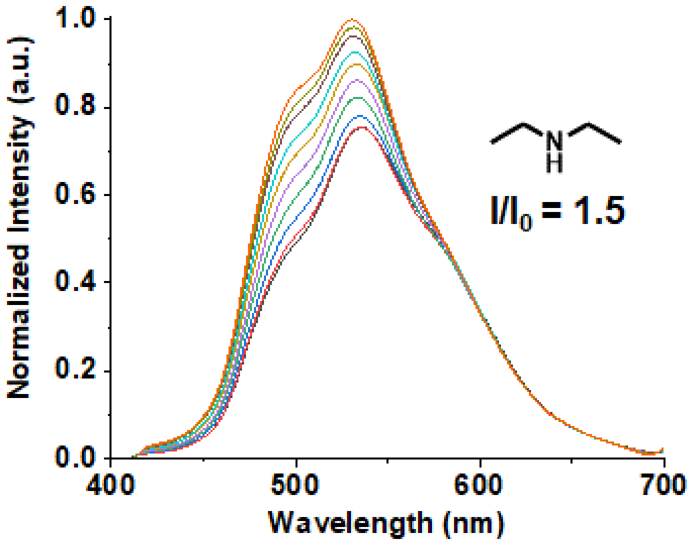

Supplementary Fig. 22. Luminescent spectra of TGH$^+$•PD in response to diethylamine. Changes observed on the emission spectrum of TGH$^+$•PD dispersed in water after exposure to diethylamine (0-10^{-6} M). The emission spectra were recorded at room temperature (λ_{ex} = 365 nm).

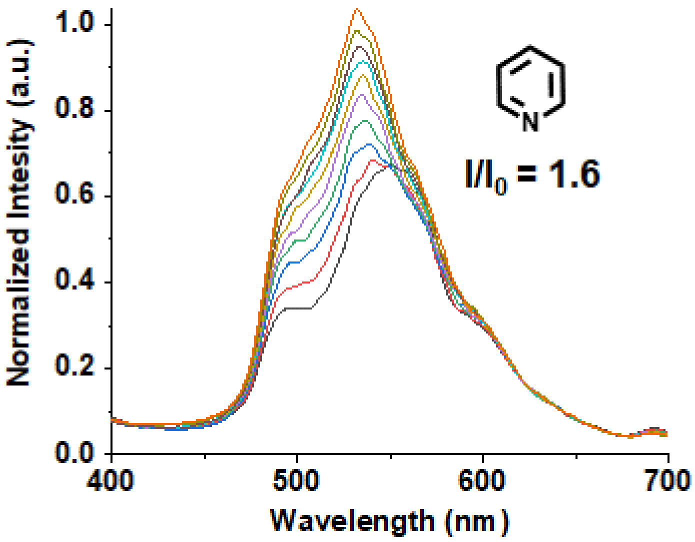

Supplementary Fig. 23. Luminescent spectra of TGH^{+}•PD in response to pyridine. Changes observed on the emission spectrum of TGH^{+}•PD dispersed in water after exposure to pyridine (0-10^{-6} M). The emission spectra were recorded at room temperature (λ_{ex} = 365 nm).

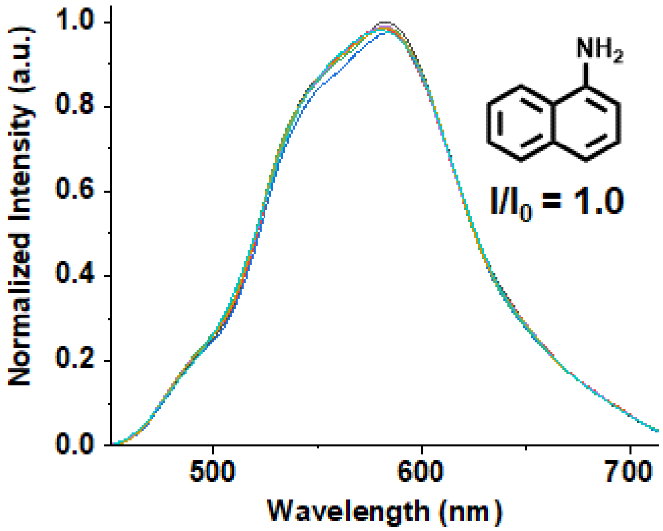

Supplementary Fig. 24. Luminescent spectra of TGH^{+}•PD in response to 1-naphthylamine. Changes observed on the emission spectrum of TGH^{+}•PD dispersed in water after exposure to 1-naphthylamine (0-10^{-6} M). The emission spectra were recorded at room temperature (λ_{ex} = 365 nm).

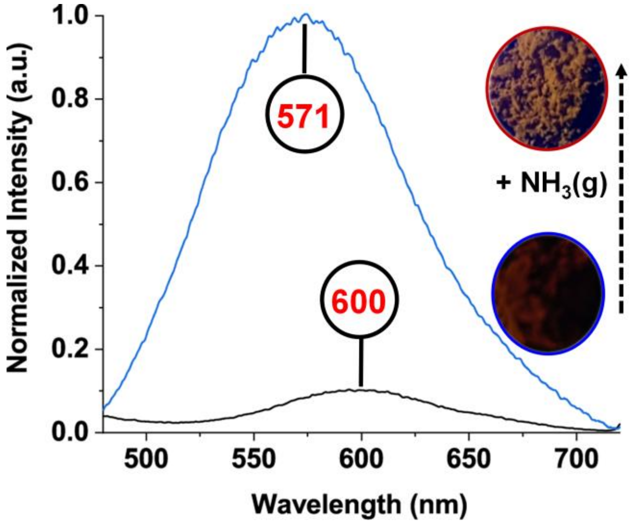

Supplementary Fig. 25. Solid state sensing. Solid-state emission spectra of activated TGH^{+}•PD COF upon exposure to NH_3 (g). The images show the changes in the luminescence response after two seconds of exposure to ammonia.

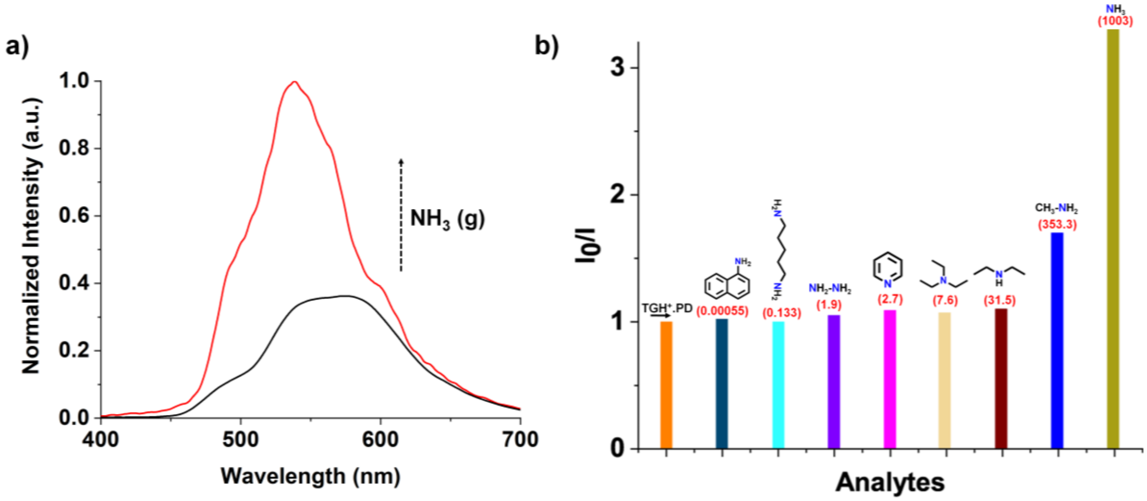

Supplementary Fig. 26. Vapor phase sensing. a) Fluorescence 'turn on' response (λ_{ex} = 365 nm) of the aqueous dispersion of TGH^{+}•PD COF upon exposure to NH_3 vapor at room temperature; b) Bar diagram representing the relative fluorescence intensity of TGH^{+}•PD in the presence of different amine vapors, where I_o is the intensity of the initial TGH^{+}•PD and I is the final intensity in response to the analyte (numbers in parentheses indicate the corresponding vapor pressure of the analytes in kilopascal at 25 °C). For vapor phase sensing, we used 5 mg of TGH^{+}•PD, dispersed in 3 mL of water.

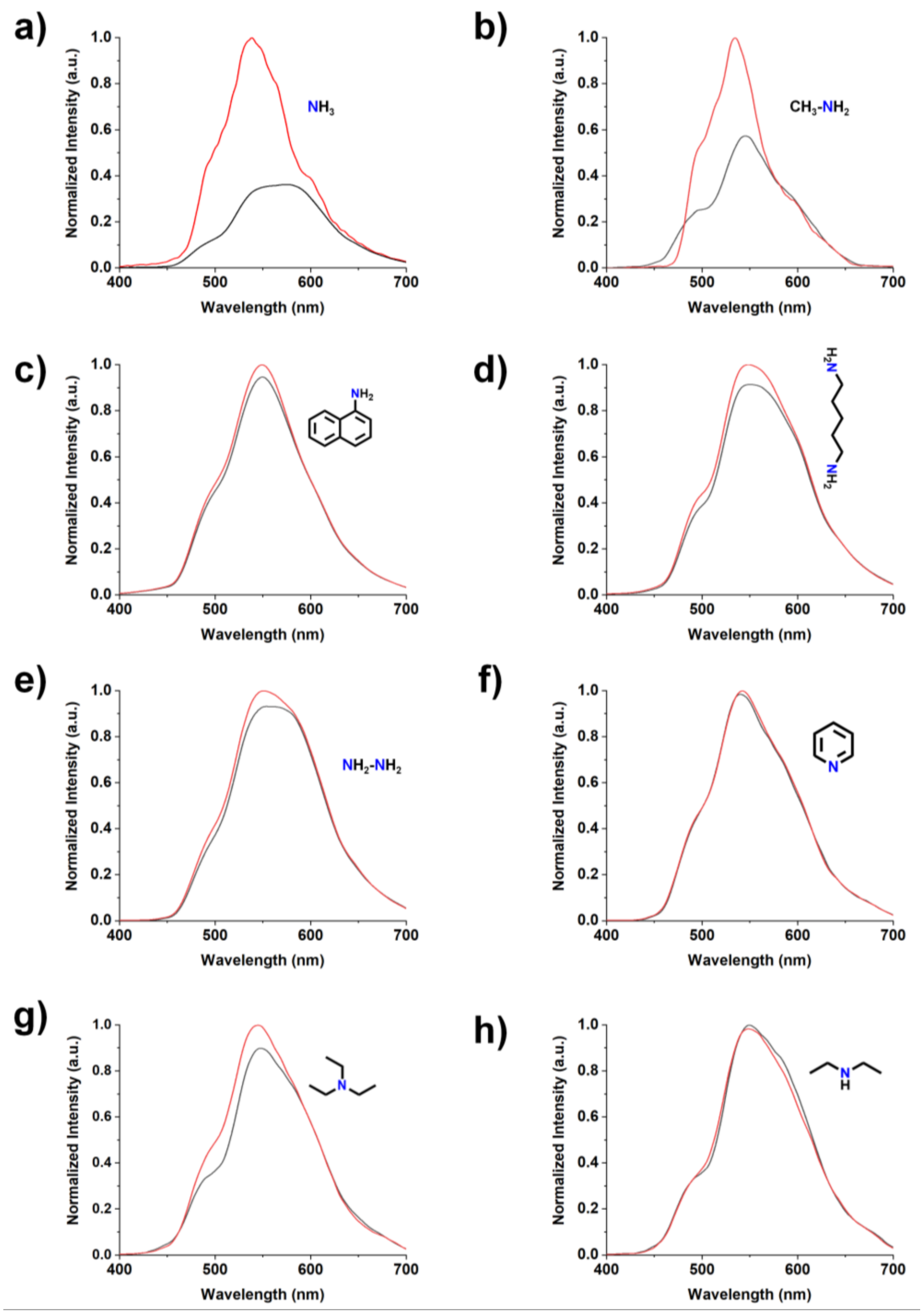

Supplementary Fig. 27. Selectivity study. Fluorescence response (λ_{ex} = 365 nm) of the aqueous dispersion of TGH^{+}•PD COF upon exposure to different amines vapor [a) ammonia, b) methylamine, c) 1-naphthylamine, d) cadaverine, e) hydrazine hydrate, f) pyridine, g) triethylamine, and h) diethylamine] at room temperature.

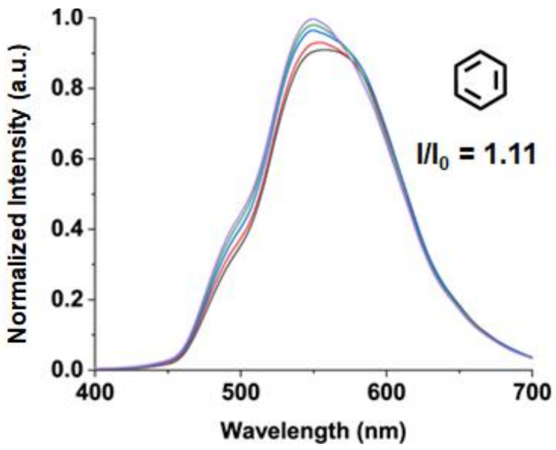

Supplementary Fig. 28. Luminescent spectra of TGH$^+$•PD in response to benzene. Changes observed on the emission spectrum of TGH$^+$•PD dispersed in water after exposure to benzene (0-10^{-6} M). The emission spectra were recorded at room temperature (λ_{ex} = 365 nm).

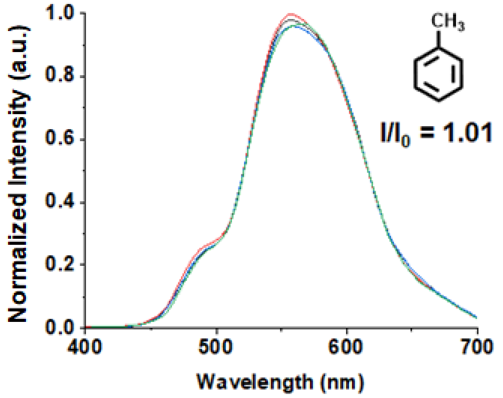

Supplementary Fig. 29. Luminescent spectra of TGH$^+$•PD in response to toluene. Changes observed on the emission spectrum of TGH$^+$•PD dispersed in water after exposure to toluene (0-10^{-6} M). The emission spectra were recorded at room temperature (λ_{ex} = 365 nm).

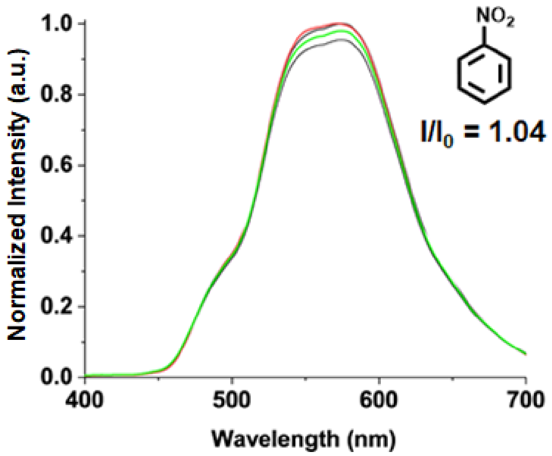

Supplementary Fig. 30. Luminescent spectra of TGH$^+$•PD in response to nitrobenzene. Changes observed on the emission spectrum of TGH$^+$•PD dispersed in water after exposure to nitrobenzene (0-10^{-6} M). The emission spectrum recorded at room temperature (λ_{ex} = 365 nm).

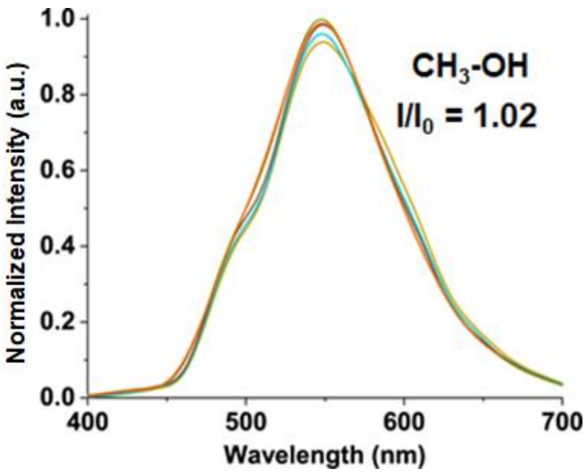

Supplementary Fig. 31. Luminescent spectra of TGH$^+$•PD in response to methanol. Changes observed on the emission spectrum of TGH$^+$•PD dispersed in water after exposure to methanol (0-10^{-6} M). The emission spectra were recorded at room temperature (λ_{ex} = 365 nm).

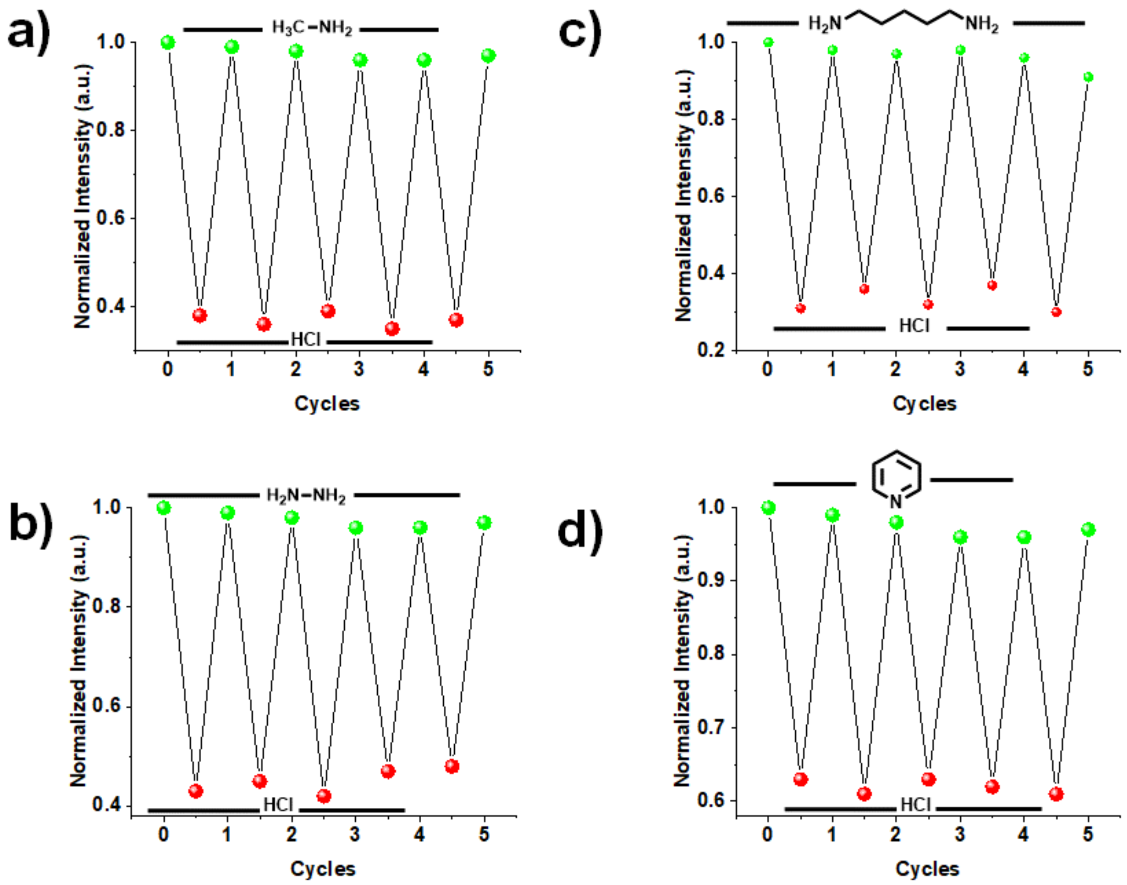

Supplementary Fig. 32. Regeneration study. Recyclability of fluorescence intensity of TGH$^+$•PD upon an alternate addition of amines (a-d) and HCl in water (λ_{ex} = 365 nm).

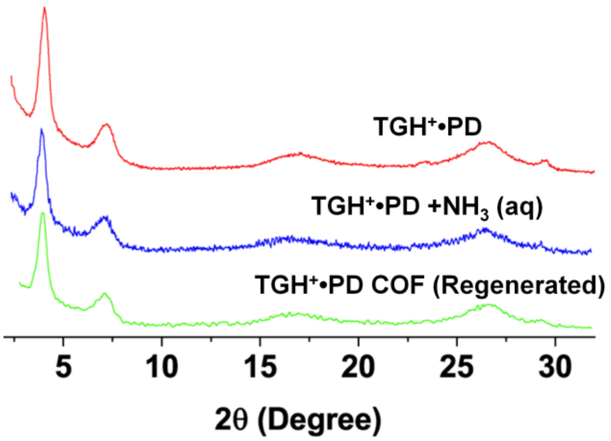

Supplementary Fig. 33. PXRD analysis after regeneration. Comparison of PXRD patterns of the TGH$^+$•PD COF before, during and after NH_3 sensing.

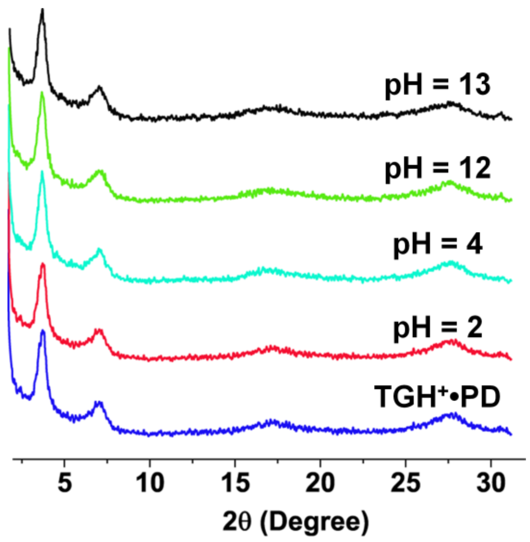

Supplementary Fig. 34. PXRD analysis at different pH. PXRD analysis of TGH^+•PD COF at different pH (2-13), Unaltered PXRD intensity of spectra indicative of their high chemical stability.

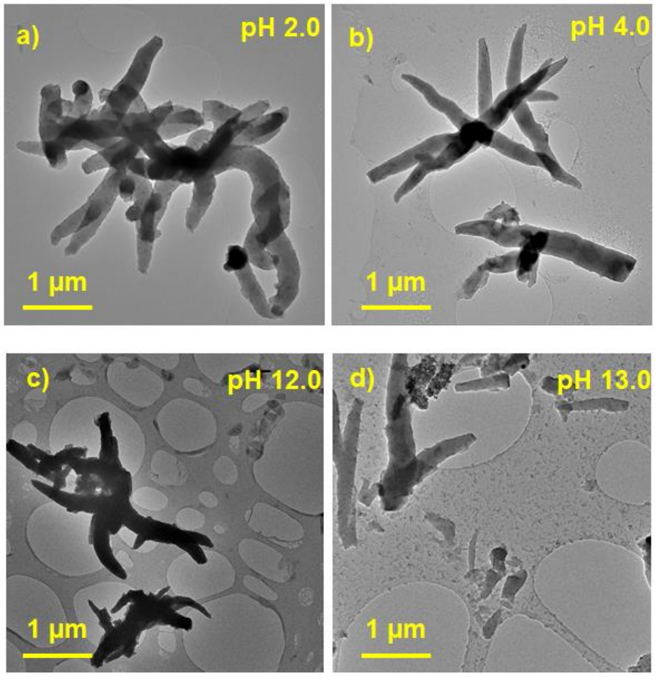

Supplementary Fig. 35. HRTEM analysis at different pH. HRTEM images of TGH^+•PD COF at different pH conditions [a) pH = 2, b) pH = 4, c) pH = 12, and d) pH = 13]. Morphology of the COF material did not show much changes at different pH.

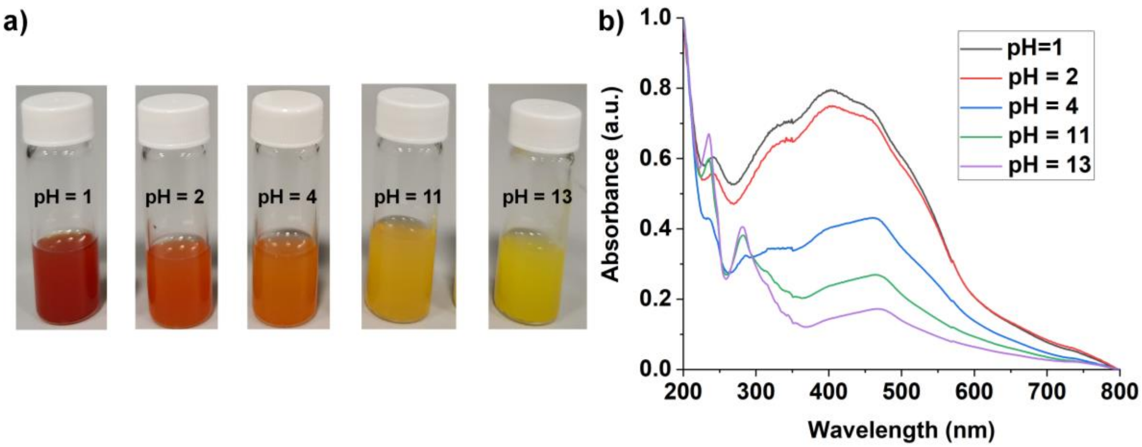

Supplementary Fig. 36. UV-vis analysis at different pH. a) Color change of TGH^{+}•PD COF in water solutions with different pH (from 1 to 13); b) UV-vis spectral changes of TGH^{+}•PD COF at different pH (1-13).

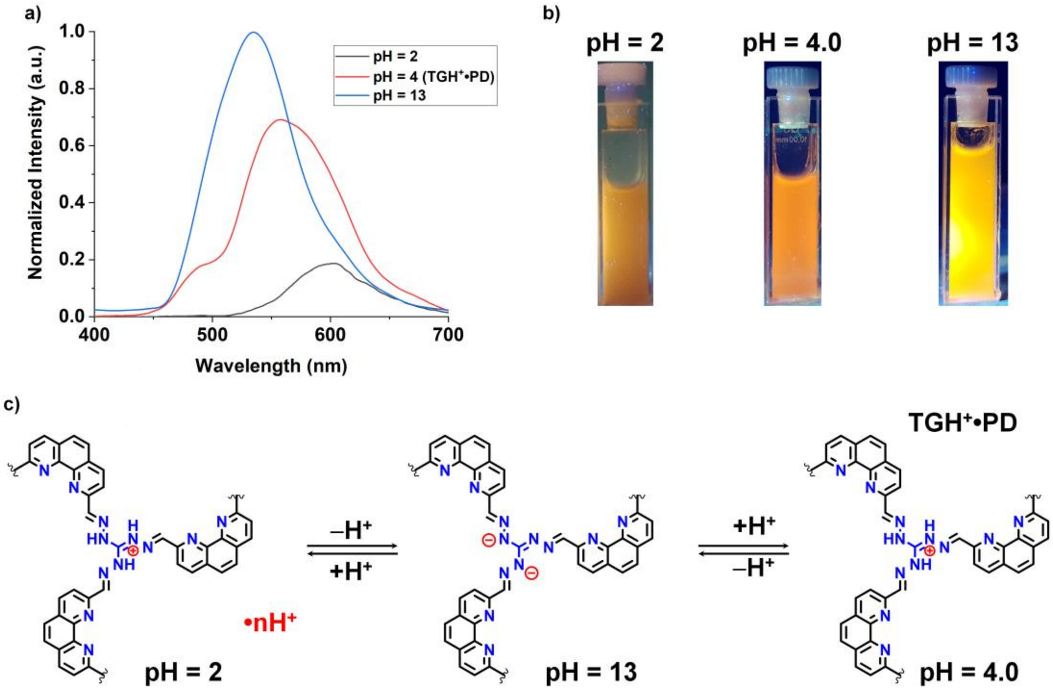

Supplementary Fig. 37. pH dependent luminescent study. a) Emission spectra of TGH^{+}•PD COF at three different pH (2, 4, 13); b) color change of emission in water. c) Mechanism of the protonation/deprotonation processes in the framework. All the spectra were recorded at room temperature.

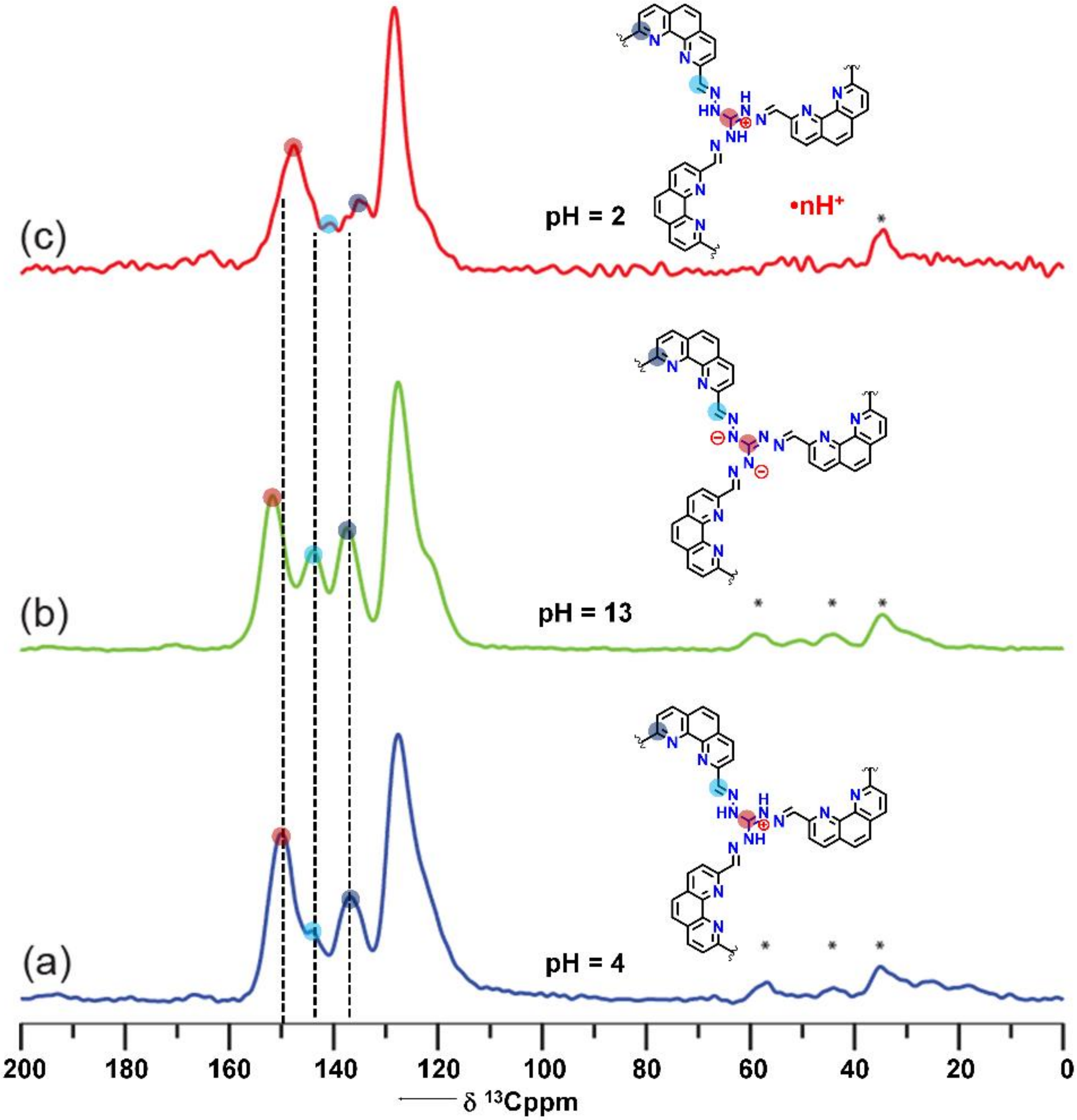

Supplementary Fig. 38. Solid state ^{13}C CP/MAS NMR analysis at different pH. ^{13}C solid-state CP/MAS NMR spectra of TGH^{+}•PD COF at a) pH = 4 (blue line, as synthesized COF), b) pH = 13 (green line), and c) pH = 2 (red line). All spectra were recorded at 25 °C.

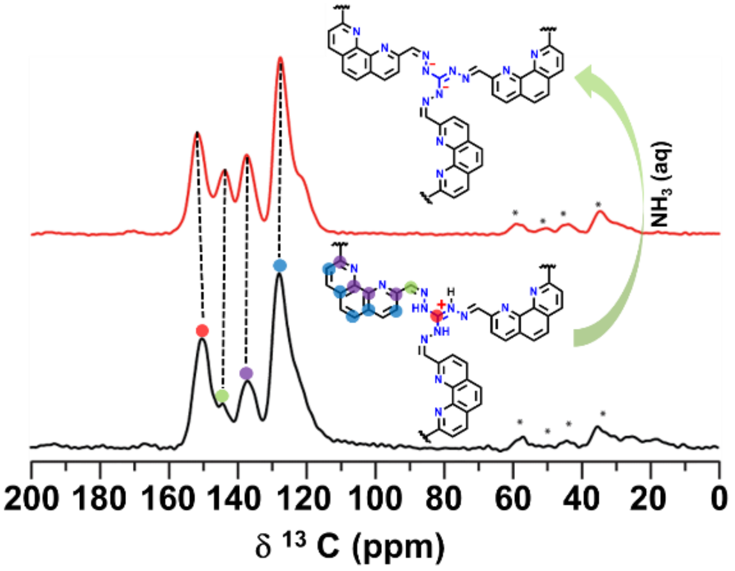

Supplementary Fig. 39. Solid state ^{13}C cross-polarization magic-angle spinning NMR spectral analysis after ammonia treatment. Stacked one-dimensional ^{13}C CP/MAS spectra of TGH^{+}•PD before (black) and after treating with ammonia (red).

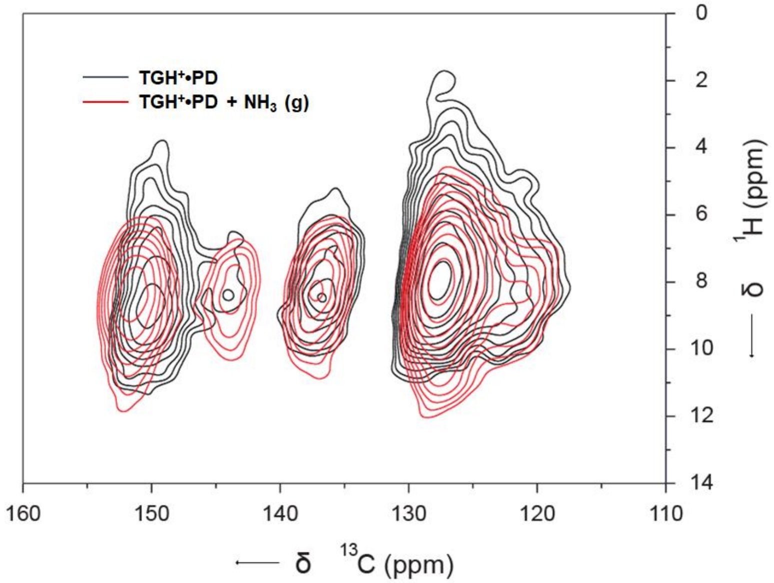

Supplementary Fig. 40. ^{1}H-^{13}C HETCOR spectra. Overlay of the two-dimensional ^{1}H-^{13}C HETCOR spectra of TGH^{+}•PD before (black) and after treating with ammonia (red).

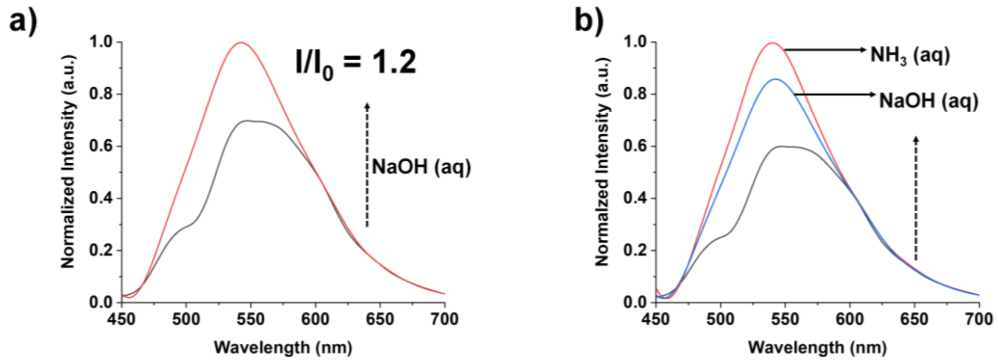

Supplementary Fig. 41. Selectivity study of TGH$^+$•PD in response to NaOH and NH_3 (aq). a) Change in the emission spectrum of TGH$^+$•PD COF dispersed in water (black line) upon addition of 10^{-2} M NaOH (red line) at room temperature; b) Fluorescence spectra of TGH$^+$•PD COF in water suspension upon addition of NaOH (aq) followed by NH_3 (aq), λ_{ex} = 365 nm.

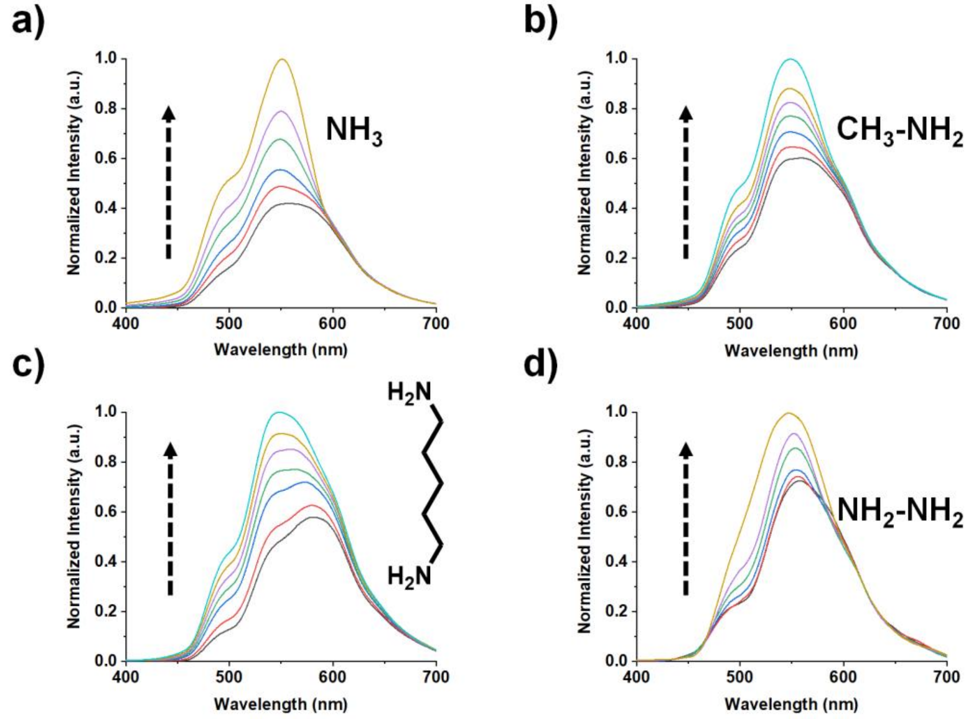

Supplementary Fig. 42. Selectivity study of TGH$^+$•PD in response to different amine analytes under anhydrous condition. Emission spectra of TGH$^+$•PD-anh COF in water dispersion when treated with different analytes [a) ammonia, b) methylamine, c) cadaverine, and d) hydrazine hydrate]. All the spectra recorded at room temperature.

Supplementary Tab .2: Comparison of I/I_0 for TGH^+•PD and TGH^+•PD-anh

Analyte	I/I_0 for TGH^+•PD	I/I_0 for TGH^+•PD-anh
Ammonia	3	2.5
Hydrazine	2.0	1.4
Methyl amine	2.6	2.0
Cadaverine	2.2	1.6

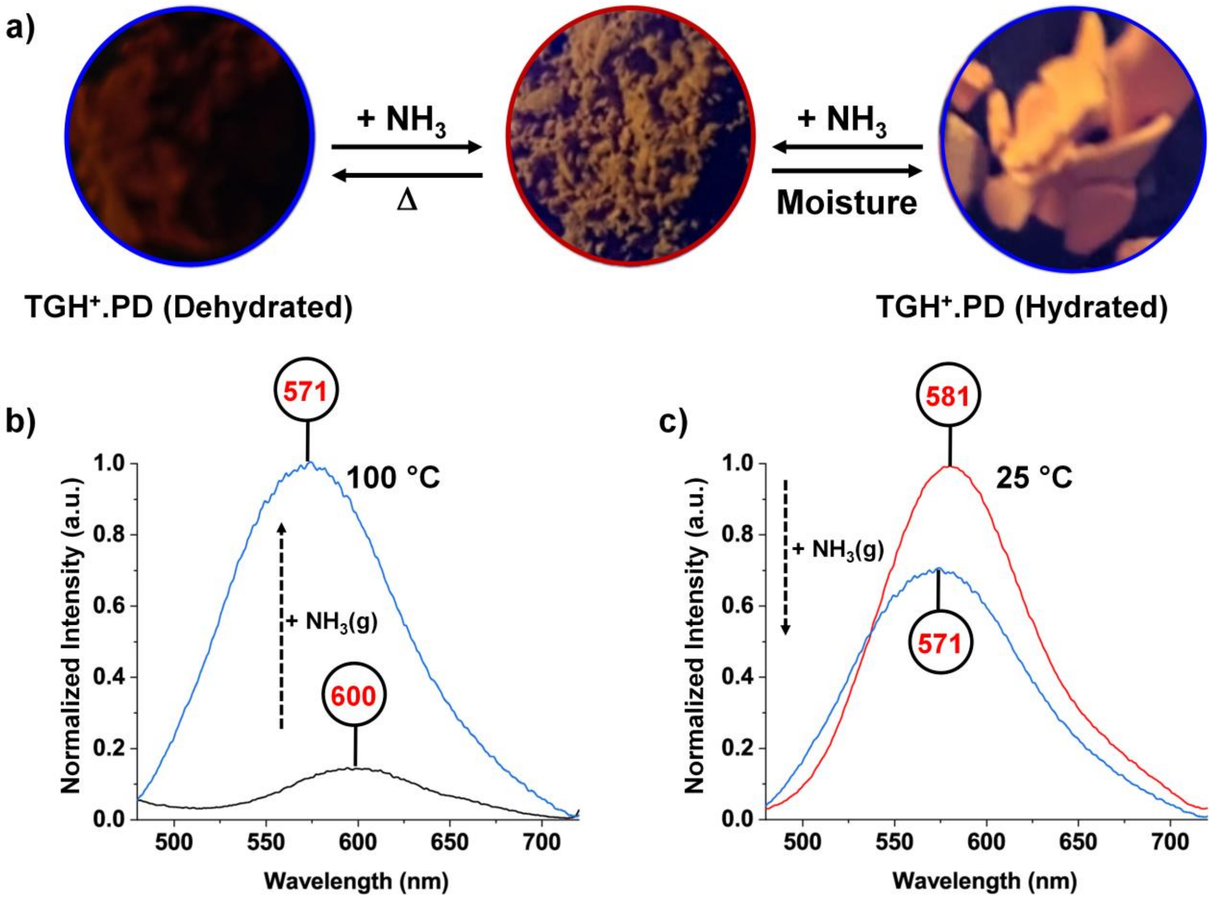

Supplementary Fig. 43. Effect of temperature on luminescent properties. a) Optical micrographs display the changes in the luminescence response of activated/hydrated TGH^+•PD COF upon exposure of ammonia for 2 sec. Solid-state emission spectra of b) activated and c) hydrated TGH^+•PD COF when exposed to NH_3 (g).

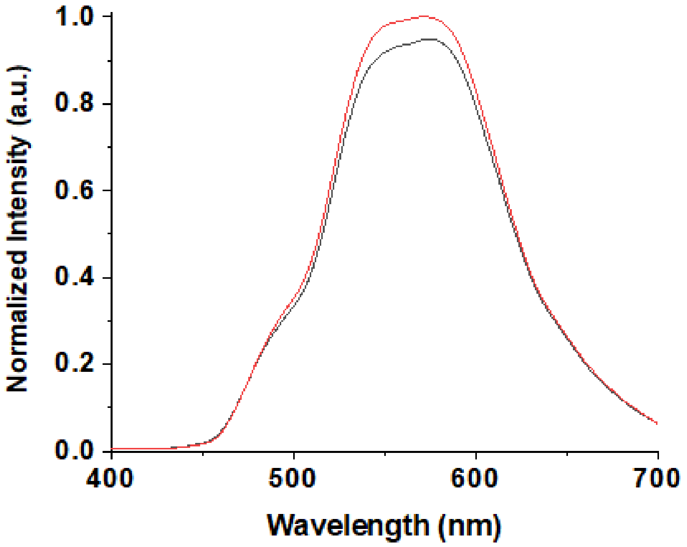

Supplementary Fig. 44. Application of TGH^{+}•PD for monitoring the quality of chicken flesh. Emission spectrum of TGH^{+}•PD in the absence (black line) and the presence of chicken flesh at 4 °C for 12 h (red line).

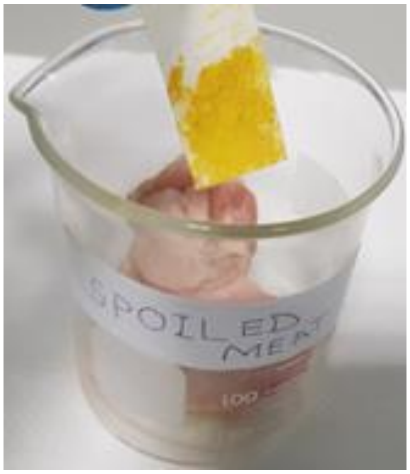

Supplementary Fig. 45. **Monitoring the chicken freshness.** Optical image of TGH^{+}•PD coated filter paper strip in the presence of spoiled chicken flesh at room temperature.

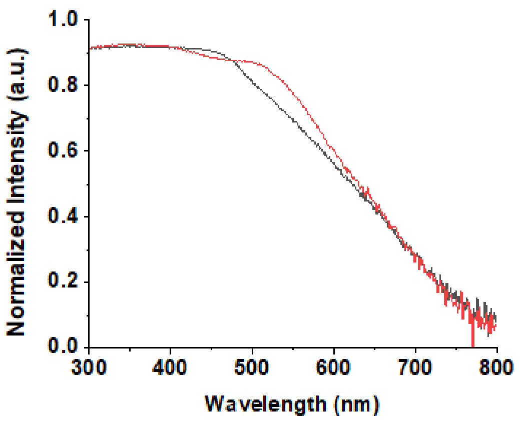

Supplementary Fig. 46. **UV-vis spectra of TGH$^+$•PD in response to ammonia.** Solid state absorbance spectra Solid-state absorbance spectra of TGH$^+$•PD in the absence (black line) and the presence of ammonia (red line) vapor at room temperature.

15. References

1 Shustova, N. B., Cozzolino, A. F., Reineke, S., Baldo, M. & Dincă, M. Selective Turn-On Ammonia Sensing Enabled by High-Temperature Fluorescence in Metal–Organic Frameworks with Open Metal Sites. *J. Am. Chem. Soc.* **135**, 13326-13329 (2013).

2 Mallick, A. *et al.* Unprecedented Ultralow Detection Limit of Amines using a Thiadiazole-Functionalized Zr(IV)-Based Metal–Organic Framework. *J. Am. Chem. Soc.* **141**, 7245-7249 (2019).

3 Mallick, A. *et al.* Solid state organic amine detection in a photochromic porous metal organic framework. *Chem. Sci.* **6**, 1420-1425 (2015).

4 Jia, R. *et al.* Amine-responsive cellulose-based ratiometric fluorescent materials for real-time and visual detection of shrimp and crab freshness. *Nat. Commun.* **10**, 795, (2019).

5 Dalapati, S., Jin, E., Addicoat, M., Heine, T. & Jiang, D. Highly Emissive Covalent Organic Frameworks. *J. Am. Chem. Soc.* **138**, 5797-5800 (2016).

6 Mani, P., Ojha, A. A., Reddy, V. S. & Mandal, S. "Turn-on" Fluorescence Sensing and Discriminative Detection of Aliphatic Amines Using a 5-Fold-Interpenetrated Coordination Polymer. *Inorg. Chem.* **56**, 6772-6775 (2017).

7 Yu, J. & Zhang, C. Fluorescent sensing for amines with a low detection limit based on conjugated porous polymers. *J. Mater. Chem. C* **8**, 16463-16469 (2020).

8 Ji, X., Yao, Y., Li, J., Yan, X. & Huang, F. A Supramolecular Cross-Linked Conjugated Polymer Network for Multiple Fluorescent Sensing. *J. Am. Chem. Soc.* **135**, 74-77 (2013).

9 Alam, P. *et al.* A Highly Sensitive Bimodal Detection of Amine Vapours Based on Aggregation Induced Emission of 1,2-Dihydroquinoxaline Derivatives. *Chem. Eur. J* **23**, 14911-14917 (2017).

10 D, B. *et al.* Rapid Visual Detection of Amines by Pyrylium Salts for Food Spoilage Taggant. *ACS Appl. Bio Mater.* **3**, 772-778 (2020).

11 Liu, Y., Xiao, Y., Shang, M., Zhuang, Y. & Wang, L. Smart fluorescent tag based on amine response for non-contact and visual monitoring of seafood freshness. *Chem. Eng. J.* **428**, 132647 (2022).

12 Jeon, S. *et al.* Amine-Reactive Activated Esters of meso-CarboxyBODIPY: Fluorogenic Assays and Labeling of Amines, Amino Acids, and Proteins. *J. Am. Chem. Soc.* **142**, 9231-9239 (2020).
13 Wang, J. *et al.* A Fluorescent Metal–Organic Framework for Food Real-Time Visual Monitoring. *Adv. Mater.* **33**, 2008020 (2021).
14 Xu, X.-Y., Lian, X., Hao, J.-N., Zhang, C. & Yan, B. A Double-Stimuli-Responsive Fluorescent Center for Monitoring of Food Spoilage based on Dye Covalently Modified EuMOFs: From Sensory Hydrogels to Logic Devices. *Adv. Mater.* **29**, 1702298 (2017).
15 Nguyen, L. H., Naficy, S., McConchie, R., Dehghani, F. & Chandrawati, R. Polydiacetylene-based sensors to detect food spoilage at low temperatures. *J. Mater. Chem. C* **7**, 1919-1926 (2019).
