# Supplementary material for: Fluorescence turn on amine detection in a cationic covalent organic framework
Source: Nat Commun. 2022 Jul 7;13:3904. doi: 10.1038/s41467-022-31393-2 (PMC9263141; doi:10.1038/s41467-022-31393-2)
Supplement: Supplementary file 2 — Description of Additional Supplementary Files [file 41467_2022_31393_MOESM2_ESM.pdf]

## Description of Additional Supplementary Files

File Name: Supplementary Movie 1
Description: Instant visual detection of ammonia
